# Quantitative acetylome and phosphorylome analysis reveals Girdin affects pancreatic cancer progression through regulating Cortactin

**DOI:** 10.18632/aging.103032

**Published:** 2020-05-05

**Authors:** Lihua Yang, Qiang Fu, Lin Miao, Quchen Ding, Xiangyu Li, Juan Wang, Guobin Jiang, Yun Wang

**Affiliations:** 1 Medical Center for Digestive Disease, The Second Affiliated Hospital of Nanjing Medical University, Nanjing 210011, China

**Keywords:** Girdin, acetylome, phosphorylome, pancreatic cancer, Cortactin

## Abstract

The actin-binding protein Girdin is involved in a variety of cellular processes, including pancreatic cancer. The objective of this study is to explore the role and the mechanism of Girdin in pancreatic cancer by quantitative acetylome and phosphorylome analysis. We firstly found that Girdin was overexpressed in pancreatic cancer tissue and increased expression of Girdin was associated with tumor size and stage of patients with pancreatic cancer. We established the shRNA knockdown of Girdin in PANC-1 and Aspc-1 cells, and we found that shGirdin inhibited proliferation, migration and invasion, and promoted apoptosis. Subsequently, we identified and quantified 5,338 phosphorylated sites in 2,263 proteins that changed in response to Girdin knockdown, and identified a similar set of Girdin-responsive acetylome data as well. Additional data revealed that down-regulation of Girdin affected Cortactin phosphorylation and acetylation, suggesting Cortactin as an important regulatory target of Girdin. Moreover, we found that overexpression of Cortactin could rescue the effect of shGirdin on proliferation, apoptosism, migration and invasion of pancreatic cancer cells. In general, our results provided new insights into the mechanisms of Girdin function including cell proliferation, migration and invasion, and offer biomarker candidates for clinical evaluation of Girdin.

## INTRODUCTION

Pancreatic cancer (PC) is a digestive tract malignant tumor with an extremely poor prognosis [1], and the median survival in terminal patients is less than 6 months [2]. In recent years, gemcitabine was the first choice of chemotherapy drugs for treating advanced pancreatic cancer [3]. But clinically, it is not ideal and does not inhibit pancreatic cancer metastasis [4]. Previous studies reported that the malignant degree of pancreatic cancer was associated with cancer stem cell (CSC) properties [5] and with abnormal expressions of a series of genes that are frequently mutated or highly up-regulated, and are correlated with drug resistance and cancer relapse [6, 7], such as aldehyde dehydrogenases (ALDHs) [8].

The ubiquitously expressed actin-binding protein Girdin/APE (Akt-phosphorylation enhancer) plays a crucial role in cytoskeleton remodeling, cell migration, and tumor metastasis. Girdin is also essential for vessel remodeling during angiogenesis [9]. It is phosphorylated and activated by the serine/threonine kinase Akt, which is a downstream target of PI3K regulation of cell polarization and migration [10]. In a variety of malignant tumor tissues, Girdin was highly expressed and was correlated with postoperative recurrence and tumor metastasis [11]. In patients with luminal-type breast cancer, Girdin expression was significantly associated with the incidence of breast cancer and lymph node metastasis [12]. Furthermore, Girdin was overexpressed in hepato-cellular carcinoma (HCC) tissues and was a target gene of the tumor suppressor miR-101. In addition, Girdin down-regulation has been shown to markedly suppress cell proliferation and migration [13]. However, the relationship between Girdin and pancreatic cancer remains unclear.

Previous studies of Girdin up- and down-regulation in cancer cells showed that Girdin was an oncogene-like protein [14, 15]. However, the proteomic response to Girdin up- and down-regulation [16, 17], including the post-translational modification (PTM) spectrogram [18, 19], remains to be investigated. During the tumorigenesis process, PTMs including phosphorylation, acetylation, or ubiquitination are dramatically changed and can play a crucial role in tumor migration or invasion. Enzymes or co-factors of PTM have important roles in modulating of transcription regulation and chromatin structure.

In this study, we explored the expression of Girdin in pancreatic cancer clinical samples and the effect of Girdin on cell proliferation, migration, invasion and apoptosis of pancreatic cancer cells. Subsequently we identified acetylome and phosphorylome changes in pancreatic cancer cell lines in response to Girdin down-regulation. We found that although Girdin is not a known nuclear protein, many of the acetylome alterations involved nuclear and mitochondria proteins. Furthermore, by bioinformatic analysis, the related Kyoto Encyclopedia of Genes and Genomes (KEGG) and Gene Ontology (GO) pathways were reported. Finally, additional data revealed that down-regulation of Girdin affected Cortactin phosphorylation and acetylation, suggesting Cortactin as an important regulatory target of Girdin.

## RESULTS

### Girdin was over-expressed in pancreatic cancer tissues and cells

**Figure 1:**
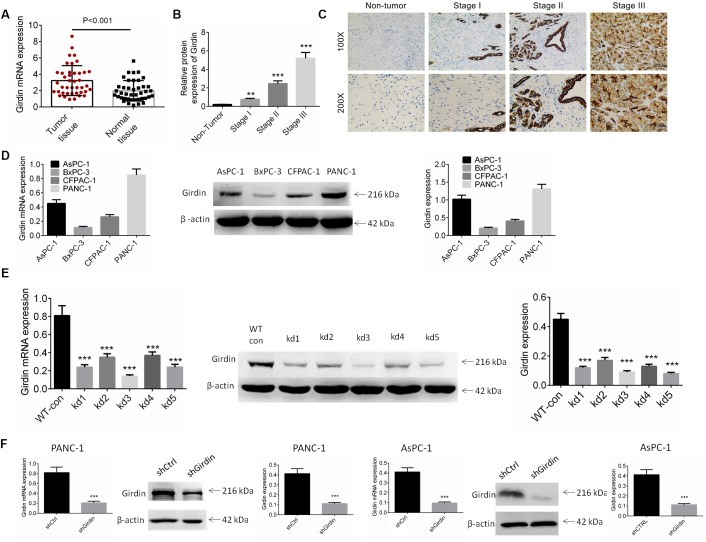
**Expression of Girdin in pancreatic cancer tissues and cell lines.** (**A**) The mRNA expression of Girdin in pancreatic cancer tissues and adjacent normal tissues was identified by RT-PCR analysis (n=41). (**B**, **C**) The protein expression of Girdin in pancreatic cancer tissues and adjacent normal tissues was examined by IHC analysis. (**D**) Girdin expression in pancreatic cancer cell lines (AsPC-1, BxPC-3, CFPAC-1 and PANC-1) was identified by western blot analysis and RT-PCR. (**E**) Girdin expression was knockdown by 5 shRNAs and Girdin expression was identified by western blot analysis. (**F**) AsPC-1 and PANC-1 cells were transfected with shGirdin plasmid, the transfection efficiency was examined by RT-PCR and western blot analysis. Data was shown as mean ± SD. ***P<0.001. The experiments were repeated 3 times.

To explore the role of Girdin in the progression of pancreatic cancer, the mRNA expression of Girdin was examined in pancreatic cancer tissues and adjacent non-tumor normal tissues. As shown in Figure 1A, the mRNA expression of Girdin was increased significantly in pancreatic cancer tissues as compared to the normal tissues (P<0.01). The protein expression of Girdin in pancreatic cancer was also identified by IHC analysis. The Figure 1B, 1C showed that Girdin was over-expressed in pancreatic cancer tissues. We subsequently identified the correlation of Girdin expression and clinicopathological characteristics of pancreatic cancer patients. As displayed in Table 1, high expression of Girdin exerted a correlation with tumor size and tumor node metastasis (TNM) stage (P<0.05).

**Table 1:** The correlation of Girdin expression and clinicopathological characteristics of pancreatic cancer patients.

**Features**	**n**	**Girdin expression**		**P value**
		**High (n=13)**	**Low (n=28)**	
Gender				
Male	22	6	16	>0.05
Female	19	7	12	
Age (years)				
≥60	25	8	17	>0.05
<60	16	5	11	
Tumor size (cm)				
≥3	18	9	9	<0.05
<3	23	4	19	
Differentiation				
Poorly differentiated	20	5	15	>0.05
Moderately and well differentiated	21	8	13	
TNM stage				
I+II	22	4	18	<0.05
III+IV	19	9	10	
Lymph node metastasis				
Yes	15	6	9	>0.05
No	26	7	19	
Distant metastasis				
Yes	21	9	12	>0.05
No	20	4	16	

TNM, tumor node metastasis.

The expression of Girdin in pancreatic cancer cell lines (AsPC-1, BxPC-3, CFPAC-1 and PANC-1) was identified by western blot and RT-PCR analysis. As shown in Figure 1D, the AsPC-1 and PANC-1 cell lines exerted higher expression of Girdin, and they were carried out for further studies. To investigate the role of Girdin in the pancreatic carcinoma cell lines, we knocked down *Girdin* expression with 5 shRNAs and shRNA-3 exhibited better efficiency (Figure 1E). An oligo targeting *Girdin* was inserted into a pLKO.1 vector, and PANC-1 and AsPC-1 cells were infected following puromycin stress screening. Our analyses of mRNA and protein expression levels with real-time quantitative PCR and western blotting, respectively, showed that Girdin was knocked down efficiently (Figure 1F).

### Girdin down-regulation regulated cell proliferation, apoptosis, migration and invasion of pancreatic cancer cells

**Figure 2:**
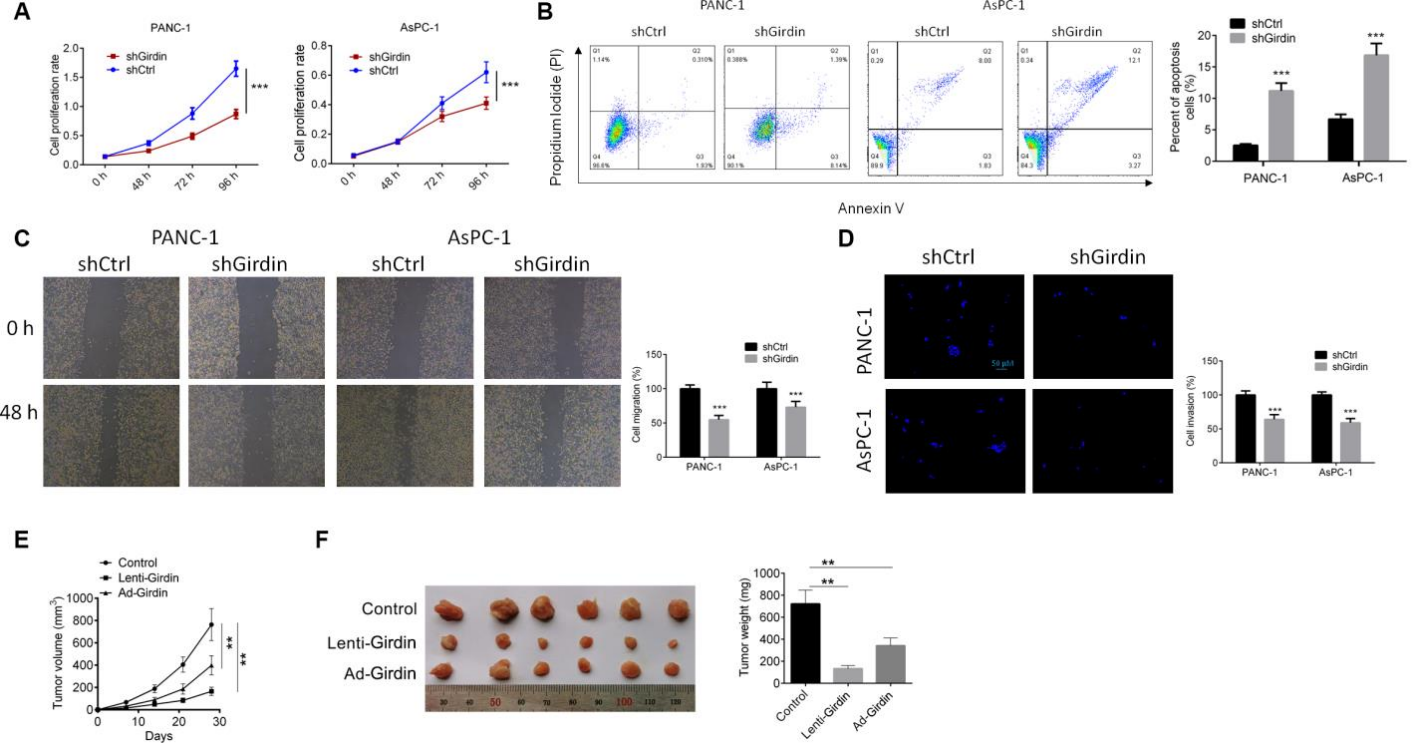
**Girdin down-regulation regulates pancreatic cancer progression *in vitro* and *in vivo*.** (**A**) Cell proliferation rates of both shCtrl and shGirdin cells were tested by CCK-8 assay. (**B**) APC/PI staining analysis to measure apoptosis levels of both shCtrl and shGirdin cells. Apoptosis levels were significantly higher in shGirdin cells. (**C**) Wound-healing assay was performed to examine the migration in PANC-1 and AsPC-1 cells with shCtrl or shGirdin transfection. (**D**) Transwell invasion assay was carried out in PANC-1 and AsPC-1 cells with shCtrl or shGirdin transfection. (**E**, **F**) Tumor growth curves were established by measuring tumor volume every 5 for 35 days after injection. Tumor weights isolated from nude mice in each treatment group were determined on day 28 after injection. Data was shown as mean ± SD. ***P<0.001. The experiments were repeated 3 times.

Having confirmed effective knockdown of Girdin in pancreatic cancer cells, we asked whether we could identify any functional associations between Girdin expression and cancer cell phenotypes. First, we examined cell proliferation by CCK8 assay. Both control pancreatic cancer cells (shCtrl) and Girdin knockdown pancreatic cancer cells (shGirdin) were seeded, and cell viability was tested after 2 days (d) and again after 4 d. At the 48-h time point, both cell lines grew at the same rate. However, after 4 d, the shGirdin cells showed significantly decreased monolayer growth compared to that of the controls (P<0.001, Figure 2A). To further investigate the biological significance of Girdin in pancreatic cancer cells, we performed an APC/PI apoptosis assay. Apoptosis rates in the shGirdin group were significantly increased compared with those of the shCtrl group (P<0.001, Figure 2B). These data indicated that Girdin down-regulation promoted cell apoptosis. We then evaluated the migratory and invasive capabilities of shGirdin cells with a wound-healing test and a transwell assay. In comparison to shCtrl cells, shGirdin cells exhibited both noticeably decreased migration (P<0.001, Figure 2C) and reduced invasion (P<0.001, Figure 2D). Together, these results suggest that Girdin regulates the metastatic capacity of pancreatic cancer, cells.

### Effects of Girdin knockdown on pancreatic tumor growth in vivo

The biological effects of Girdin on pancreatic cancer development were evaluated in a xenograft mouse model. PANC-1 cells transfected with shCtrl or shGirdin were implanted subcutaneously into nude mice. Then tumor growth was evaluated every 5 days. Girdin knockdown significantly delayed tumor growth *in vivo* (P<0.01, Figure 2E, 2F). At 5 weeks postimplantation, the nude mice were sacrificed, and tumors were harvested and weighed. Girdin knockdown significantly decreased the tumor size and weight (P<0.01, Figure 2G, 2H).

### Acetylome quantification

Next, we sought to identify the mechanism(s) by which Girdin regulates cell proliferation, migration, and invasion. Both acetylation and phosphorylation were performed to qualify the proteome acetylation changes in shGirdin knockdown PANC-1 cells LC-MS/MS. For acetylome quantification, 2,927 lysine acetylation sites in 1,196 protein groups were identified, among which 2,873 sites in 1,183 proteins were quantified (Supplementary Table 1). When setting quantification ratio of >1.5 as up-regulated threshold and <0.67 as down-regulated threshold, 93 lysine acetylation sites in 80 proteins were quantified as up-regulated targets and 266 lysine acetylation sites in 196 proteins were quantified as down-regulated targets.

### Biological analysis of acetylome

**Figure 3:**
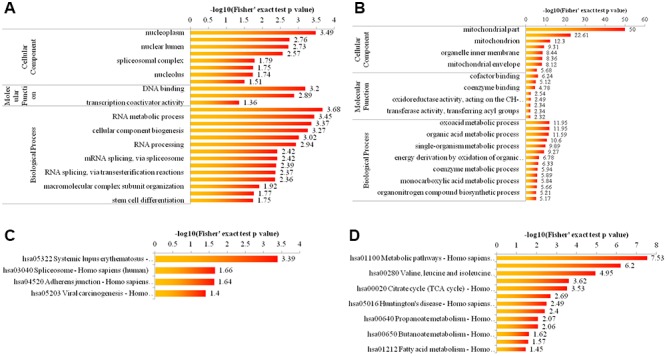
**Bioinformatic analysis of acetylome quantification.** (**A**, **B**) The enrichment of up- and down-regulated proteins in GO including cellular component analysis, biological process analysis, and molecular function analysis. (**C**, **D**) The enrichment of up- and down-regulated proteins in KEGG pathways.

To elucidate the cellular functions regulated by Girdin, we examined the acetylome data enriched for GO categories and KEGG pathway. As shown in Figure 3A, 3B for GO enrichment, the upregulated proteins were highly enriched in nucleoplasm, DNA binding and nucleic acid metabolic process (Figure 3A), and the downregulated proteins were highly enriched in mitochondrial part, cofactor binding, and oxoacid metabolic process (Figure 3B). As displayed in Figure 3C, 3D for KEGG pathway enrichment, the upregulated proteins were highly enriched in hsa05322 Systemic lupus erythematosus-Homo sapiens (human) (Figure 3C), and the downregulated proteins were highly enriched in hsa01100 Metabolic pathways-Homo sapiens (human) (Figure 3D).

### Quantification of phosphorylation

Using SILAC labeling and affinity enrichment followed by high-resolution LC-MS/MS analysis, quantitative phosphorylome analysis was performed in pair of PANC-1 lines. Altogether, 5,468 phosphorylation sites in 2,317 protein groups were identified, among which 5,338 sites in 2,263 proteins were quantified (Supplementary Table 2). When setting quantification ratio of >1.5 as up-regulated threshold and <0.67 as down-regulated threshold, 657 phosphorylation sites in 474 proteins were quantified as up-regulated targets and 404 phosphorylation sites in 307 proteins were quantified as down-regulated targets.

### Biological analysis of phosphorylation

**Figure 4:**
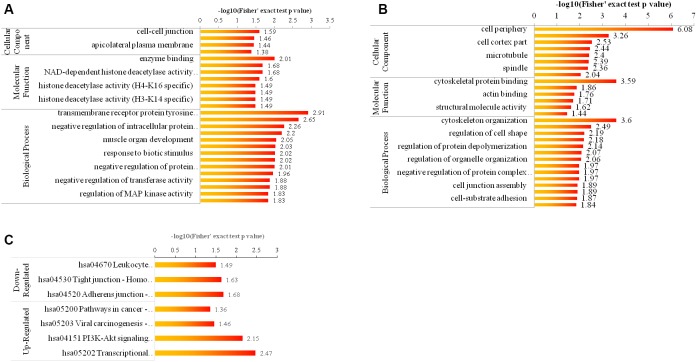
**Bioinformatic analysis of the quantified phosphorylome.** (**A**, **B**) The enrichment of up- and down-regulated proteins in GO including cellular component analysis, biological process analysis, and molecular function analysis. (**C**) The enrichment of up- and down-regulated proteins in KEGG pathways.

An enrichment-based clustering analysis was carried out to compare the function of corresponding proteins with up- and down- regulated phosphorylation level. As shown in Figure 4A, 4B for GO enrichment, the upregulated proteins were highly enriched in cell-cell junction, enzyme binding and transmembrane receptor protein tyrosine kinase signaling pathway (Figure 4A), and the downregulated proteins were highly enriched in cell periphery, cytoskeletal protein binding, and cytoskeleton organization (Figure 4B). As displayed in Figure 4C for KEGG pathway enrichment, the down-regulated proteins were highly enriched in hsa04670 Leukocyte transendothelial migration-Homo sapiens (human) (Figure 4C), and the up-regulated proteins were highly enriched in hsa05200 Pathways in cancer-Homo sapiens (human) (Figure 4C).

### Crosstalk between quantitative phosphorylome and acetylome

**Figure 5:**
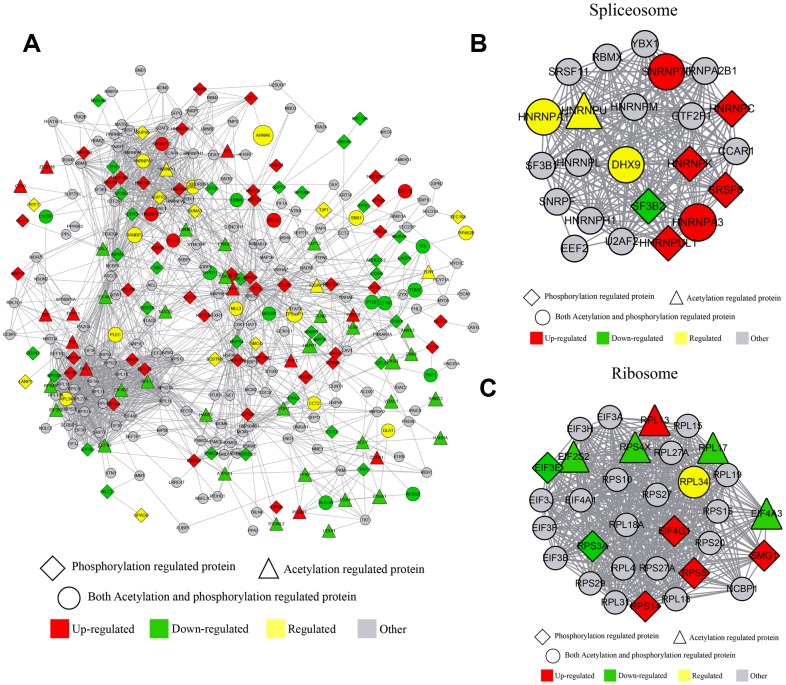
**Crosstalk between quantitative phosphorylome and acetylome.** (**A**) The protein-protein interaction network of phosphorylated and acetylated proteins. (**B**) The protein-protein interaction network clustered in the RNA spliceosome. (**C**) The protein-protein interaction network clustered in ribosome.

Several proteins were phosphorylated and acetylated at different sites and at different conditions; knocking down of Girdin affected a series of protein phosphorylation and acetylation simultaneously. By comparing the acetylome and phosphorylome data, we identified more than 300 proteins that undergo acetylation and phosphorylation by the same protein at different sites. To reveal the crosstalk between these two PTM in detail, the protein-protein interaction network was established as shown in Figure 5. The global overview of protein-protein interaction networks was obtained (Figure 5A); then, these two groups of data were classified into multiple biological processes. Thus, it realized that ribosome and RNA spliceosome participated in Girdin-regulated biological functions (Figure 5B, 5C), in which some essential proteins including HNRNPA1, DXH9, and HNRNPA3 were identified and whose phosphorylation and acetylation level were adjusted by Girdin protein expression.

### Girdin knockdown affects Cortactin phosphorylation and acetylation

**Figure 6:**
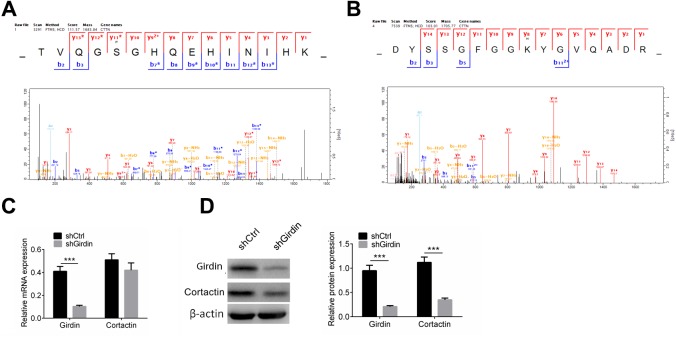
**Cortactin protein level is decreased when knocking down Girdin.** (**A**, **B**) Cortactin phosphorylation and acetylation. Cortactin was phosphorylated at S47 (**A**) and acetylated at K161 (**B**). (**C**) Expression of *Girdin* and *Cortactin* transcripts following shRNA knockdown of Girdin in PANC-1 cells was assessed by qPCR. (**D**) Western blotting results showed Girdin and Cortactin protein expression levels in shCtrl cells and shGirdin cells, with GAPDH loading control. Data was shown as mean ± SD. ***P<0.001. The experiments were repeated 3 times.

We confirmed that knocking down Girdin affected cell proliferation, migration and invasion, and further confirmed that the acetylation and phosphorylation of numerous proteins were changed in this gene control process. We further discovered the phosphorylation and acetylation of one protein, Cortactin, which contributes to the organization of the actin cytoskeleton and cell shape and plays a role in cell migration was dramatically affected by knocking down Girdin. To be quantitatively identified, 11 phosphorylation sites and seven acetylation sites were downregulated by decreasing the expression of Girdin protein. As shown in Figure 6A, 6B, S47 and K161 was phosphorylated and acetylated respectively, both modifications were decreased when Girdin was knocked down. As proteins’ phosphorylation and acetylation exchange were able to affect its stability and degradation, we assumed that knocking down Girdin could affect multiple cellular functions through regulating Cortactin expression.

We then tested *Cortactin* mRNA levels in Girdin knockdown PANC-1 cells by RT-PCR and western blot analysis. The mRNA expression of *Cortactin* did not change (Figure 6C). As shown in Figure 6D, Cortactin protein was decreased significantly when Girdin was knocked down using shRNA. These results suggested that Girdin may regulate cell proliferation, migration, and invasion by regulating Cortactin expression.

### Cortactin overexpression reversed the effect of shGirdin on PANC-1 cells.

**Figure 7:**
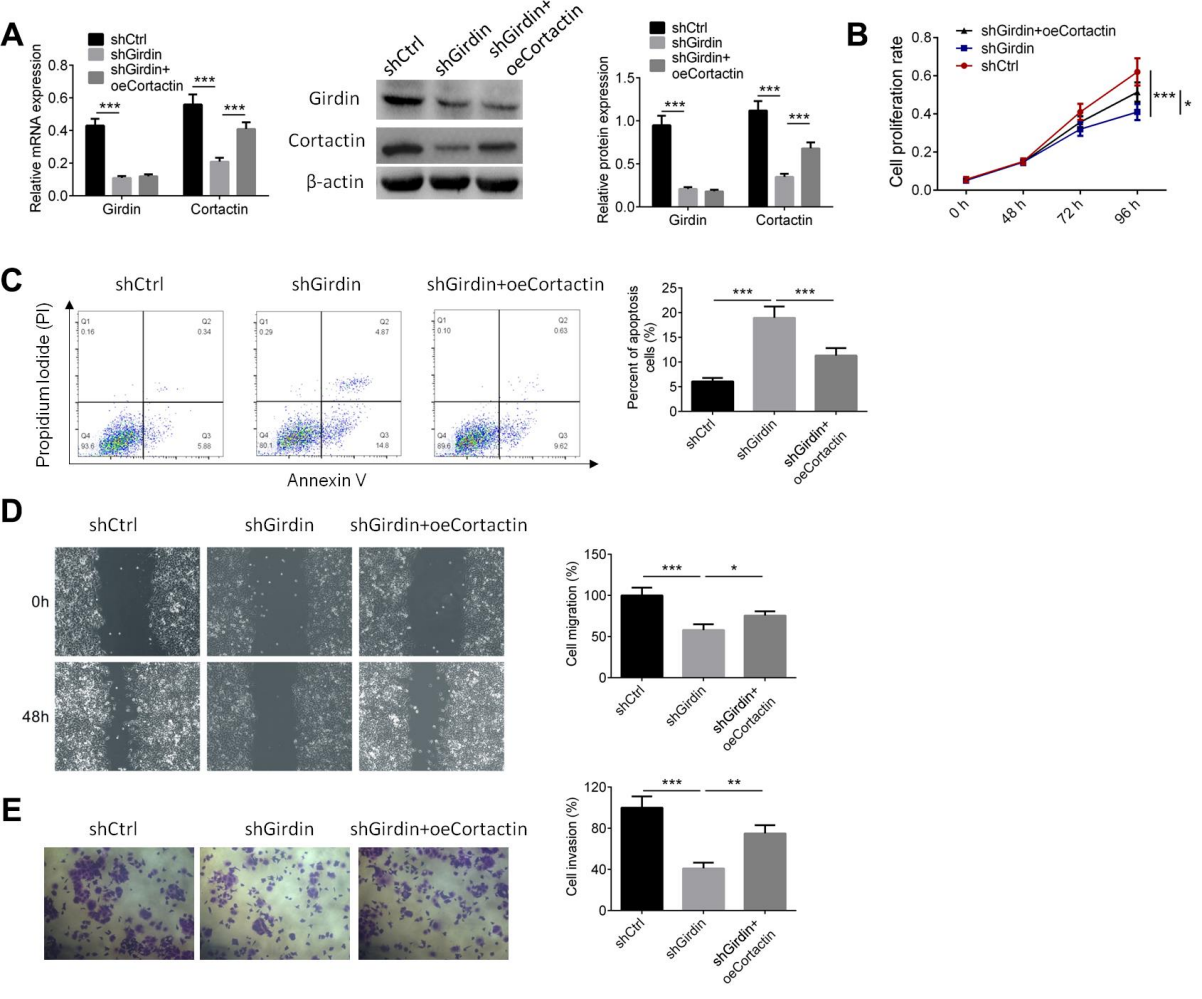
**Cortactin over-expression reversed the effect of shGirdin on PANC-1 cells.** PANC-1 cells were transfected with shNC, shGirdin+oeNC, and shGirdin+oeCortactin. (**A**) RT-PCR and western blot analysis were employed to detect the expressions of Girdin and Cortactin. (**B**–**E**) Cell proliferation, apoptosis, migration and invasion was identified by CCK8, APC/PI staining, wound healing, and transwell assays, respectively. Data was shown as mean ± SD. *P<0.05, **P<0.01, ***P<0.001. The experiments were repeated 3 times.

To further identify the mechanism by which Girdin regulated the proliferation and invasion of pancreatic cancer cells, PANC-1 cells were transfected with shNC, shGirdin+oeNC, and shGirdin+oeCortactin. RT-PCR and western blot analysis were employed to detect the expressions of Girdin and Cortactin, and the results showed that Cortactin overexpression showed no effect on Girdin expression (Figure 7A). Cell proliferation, apoptosis, migration and invasion were identified by CCK8, APC/PI staining, wound healing, and transwell assays, respectively. As shown in Figure 7B–7E, shGirdin inhibited cell proliferation, induced cell apoptosis and suppressed cell migration and invasion (P<0.001), while overexpression of Cortactin reversed the anti-tumor effect of shGirdin on PANC-1 cells (P<0.05).

## DISCUSSION

Girdin is involved in a variety of cellular processes including correct neuron positioning, dendritic development, and cell migration [20]. It is also essential in tube formation and VEGF-dependent migration of endothelial cells [9]. Girdin is phosphorylated by the serine/threonine protein kinase Akt; however, downstream regulatory role(s) for Girdin remains unknown [21]. The goal of this study was to characterize the effect and the underlying mechanism of Girdin on the proliferation, migration, and invasion of pancreatic cancer cells, which could eventually provide a model for developing targeted therapies for pancreatic cancer. From collecting and analysis of the clinical data of pancreatic cancer patients, we found that Girdin was overexpressed in pancreatic cancer and higher expression of Girdin was associated with tumor size and TNM stage. Previous studies revealed that Girdin regulated cell activities in lung cancer and breast cancer [22, 23]. By a series of functional experiments *in vitro* and *in vivo*, we also found that down-regulation of Girdin suppressed cell proliferation, migration and invasion, while promoted cell apoptosis. Over-expression of Girdin facilitated tumor growth *in vivo*. The results raised the possibility that Girdin inhibitors could be candidates of pancreatic cancer drugs.

We subsequently performed the quantitative acetylome and phosphorylome analysis by using SILAC labeling and affinity enrichment followed by high-resolution LC-MS/MS analysis, because these are two important mechanisms of protein regulation [24, 25]. We identified 2,927 acetylated lysine sites in 1,196 proteins, of which we quantified 2,873 acetylated lysine sites in 1,183 proteins. Enrichment analysis of differentially expressed proteins revealed that upregulated proteins were highly enriched in nucleoplasm, DNA binding, nucleic acid metabolic process and hsa05322 systemic lupus erythematosus, while upregulated proteins were highly enriched in mitochondrial part, cofactor binding, oxoacid metabolic process, and hsa01100 Metabolic pathway. These pathways are closely related cell phenotype of malignant tumors [26, 27]. By quantitative phosphorylome analysis, 5,468 phosphorylation sites in 2,317 protein groups were identified, among which 5,338 sites in 2,263 proteins were quantified. the upregulated proteins were highly enriched in cell-cell junction, enzyme binding, transmembrane receptor protein tyrosine kinase signaling pathway and hsa04670 leukocyte transendothelial migration pathway, and the downregulated proteins were highly enriched in cell periphery, cytoskeletal protein binding, and cytoskeleton organization and hsa05200 pathways in cancer pathways. A lot of studies report that cell-cell junction [28], tyrosine kinase signaling pathway [29], cytoskeletal protein binding [30], and cytoskeleton organization pathways are involved in the regulation of cancer cell growth and invasion [31]. These results indirectly showed that Girdin regulated cell activities of pancreatic cancer cell through regulating these pathways.

Crosstalk between quantitative phosphorylome and acetylome revealed that ribosome and RNA spliceosome participated in Girdin-regulated biological functions, and some essential proteins including HNRNPA1, DXH9, and HNRNPA3 were identified and whose phosphorylation and acetylation level were adjusted by shGirdin. We further discovered the phosphorylation and acetylation of Cortactin, which contributed to the organization of the actin cytoskeleton and cell shape [32], and 11 phosphorylation sites and 7 acetylation sites of Cortactin were downregulated by decreasing the expression of Girdin protein. Several studies show that Cortactin plays a role in cell migration and invasion in colorectal cancer, gastric cancer, lung cancer and pancreatic cancer [33]. Subsequently, the expression of Cortaction in pancreatic cancer cells with shGirdin transfection was examined, and the results showed that protein expression of Cortactin was decreased while the mRNA expression of Cortaction showed no significant change. Finally the rescue experiments were carried out, the results displayed that overexpression of Cortactin significantly abolished the anti-tumor effect of shGirdin on pancreatic cancer cells. The results revealed that Girdin affected the cell proliferation, migration, invasion and apoptosis through regulating the acetylation and phosphorylation of Cortactin.

Overall, our results investigates the acetylation and phosphorylation regulated by Girdin, which provides new insights into the molecular mechanism of Girdin function in pancreatic cancer cells including cell proliferation, migration and invasion, and offers potential biomarker candidates for the clinical evaluation of Girdin and Cortactin.

## MATERIALS AND METHODS

### Patient and tissue samples

Paired pancreatic cancer tissues (n=41) and matched peritumor samples were obtained from a tissue bank of samples collected from patients (age range, 41-79 years; mean age, 58 years; male:female ratio, 22:19) that underwent surgical treatment between January 2010 and December 2016 at the Second Affiliated Hospital of Nanjing Medical University. This study did not include patients that had received radiotherapy and/or immunotherapy prior to or following surgical treatment. Tissue samples were snap frozen in liquid nitrogen immediately following surgical resection and stored at -80°C. Written informed consent was obtained from all patients. The study protocol conformed to the ethical guidelines of the 1975 Declaration of Helsinki, and was approved by the Second Affiliated Hospital of Nanjing Medical University.

### Cell culture

AsPC-1, BxPC-3, CFPAC-1 and PANC-1 cell lines were purchased from the Chinese Academy of Sciences. Cells were cultured in RPMI-1640 medium supplemented with 10% fetal bovine serum (FBS) and 1% penicillin/streptomycin. Cells were cultured in a humidified tissue culture incubator with 5% carbon dioxide (CO_2_) at 37°C.

### Adenoviral infection

The adenovirus vector expressing shGirdin and the control blank adenovirus were purchased from Jikai (Shanghai). We found that a multiplicity of infection (MOI) of 50 was an optimal infection dose for adenovirus-mediated gene transfer in PANC-1 and AsPC-1 cells. Thus, PANC-1 and AsPC-1 cells were infected with AdV-shGirdin (shGirdin) or AdV-shCtrl (shCtrl) at an MOI of 50 throughout the study.

PANC-1 and AsPC-1 cells were plated in 6-well plates at 6 × 10^5^ cells per well and subsequently infected with eithershGirdin or shCtrl in culture medium (RPMI-1640 medium supplemented with 10% fetal bovine serum). After 24 hours (h) of infection, the shGirdin- or shCtrl-infected cells were collected for subsequent experiments.

### Proteomic quantification of phosphorylation

Using SILAC labeling and affinity enrichment followed by high-resolution LC-MS/MS analysis, quantitative phosphorylome analysis was performed in pair of human cell lines. Altogether, 5,468 phosphorylation sites in 2,317 protein groups were identified, among which 5,338 sites in 2,263 proteins were quantified. When setting quantification ratio of >1.5 as up-regulated threshold and <0.67 as down-regulated threshold, 657 phosphorylation sites in 474 proteins were quantified as up-regulated targets and 404 phosphorylation sites in 307 proteins were quantified as down-regulated targets. Intensive bioinformatic analysis was then carried out to annotate those quantifiable targets, including protein annotation, functional classification, functional enrichment, functional enrichment-based cluster analysis, etc (Supplementary File 1). Based on the results, further studies following the quantitative proteome analysis were suggested.

### Proteomic quantification of lysine acetylation analysis

Using SILAC labeling and affinity enrichment followed by high-resolution LC-MS/MS analysis, quantitative lysine acetylome analysis was performed in pair of human cell lines. Altogether, 2,927 lysine acetylation sites in 1,196 protein groups were identified, among which 2,873 sites in 1,183 proteins were quantified. When setting quantification ratio of >1.5 as up-regulated threshold and <0.67 as down-regulated threshold, 93 lysine acetylation sites in 80 proteins were quantified as up-regulated targets and 266 lysine acetylation sites in 196 proteins were quantified as down-regulated targets. Intensive bioinformatic analysis was then carried out to annotate those quantifiable targets, including protein annotation, functional classification, functional enrichment, functional enrichment-based cluster analysis, etc (Supplementary File 2). Based on the results, further studies following the quantitative proteome analysis were suggested.

### RNA extraction

After 24 h of infection, the shGirdin- or shCtrl-infected cells were harvested into 1.5 ml tubes and were spun down at 1,000 revolutions per minute (rpm) for 3 minutes (min). The pellet was resuspended and washed with 1ml PBS. Cells were spun down at 1,000 rpm for 3 min, then lysed in 1 ml of Trizol and homogenized. The homogenized solution was incubated at room temperature for five min. Next, 200μl chloroform was added to the homogenate, vortexed for 15 seconds (sec), and incubated at room temperature for 5 min. Samples were centrifuged at 12,000 rpm for 15 min at 4°C. Following centrifugation, the supernatant (aqueous phase) was transferred to a fresh tube and incubated with 500 μl isopropanol for 10 min at room temperature. Samples were centrifuged at 12,000 rpm for 15 min at 4°C. Following centrifugation, the supernatant was removed and the RNA pellet was washed with 1 ml of 75% ethanol (12,000 *g* for 5 min at 4°C). The supernatant was removed, and the pellet was air dried for 10-15 min. The RNA pellet was then re-dissolved in 20-50 μl of DEPC water.

### Reverse transcription PCR

Total RNA was extracted with Trizol reagent followed by a standard phenol-chloroform extraction protocol. First-strand complementary DNA (cDNA) was synthesized from the extracted total RNA. Total RNA (500 ng) was transcribed with 5X PrimeScript MasterMix. 2 ul 5X PrimeScript Master Mix was added to the tube and RNase-free water was added, up to 10 μl. After gently mixing the reaction solution, the reverse-transcription reaction was carried out according to the manufacturer’s protocol. The PCR conditions were: 37°C for 15 min (reverse transcription), 85°C for 5 sec (heat inactivation of reverse transcriptase), and 4°C. The resulting cDNA was used for quantitative real-time RT-PCR (qRT-PCR).

### Quantitative real time PCR

The primer sequences used for the amplification were as follows.

Girdin Forward Primer: 5'-AGGCTTCAGATCCAAGCAGT -3',

Girdin Reverse Primer: 5'- TGTTCTGCCACTATCAAGGC -3';

Cortactin Forward Primer: 5'- AAAGCTTCAGCAGGCCAC -3',

Cortactin Reverse Primer: 5'- TTGGTCCTGTTTCAAGTTCC -3';

GAPDH Forward Primer: 5'-AAGGTGGTGAAGCAGGCGTCG-3', and

GAPDH Reverse Primer: 5'-AATGCCAGCCCCAGCGTCAAAG-3'.

GAPDH was used as an internal control to normalize for differences in the amount of total RNA in each sample. The cDNA was used as a template in real-time PCR (RT-PCR) reactions. PCR reactions were prepared according the SYBR green supermix protocol. RT-PCR reactions were run on real-time PCR detection systems (Bio-Rad). PCR amplification cycles were preceded by a 10-min denaturation step at 95°C followed by 40 cycles of 95°C for 15 sec, 60°C for 60 sec respectively, and 95°C for 15 sec, 60°C for 1 min, 95°C for 15 sec. Melt curves were generated after PCR amplification. The threshold cycles were used to calculate relative expression levels according to the 2^-∆∆Ct^ method. Relative mRNA expression levels were normalized to the level of GAPDH.

### Western blotting

PANC-1 and AsPC-1 cells were plated in 6-well plates at 6 × 10^5^ cells per well, and were subsequently treated with either shGirdin (50 MOI) or shCtrl (50 MOI) in culture medium. Culture medium was replaced after 48 h. After another 48 h of treatment, the shGirdin or shCtrl treated PANC-1 cells were harvested in 1.5 ml tubes. Then cells were washed with PBS and the pellet was resuspended with 100 μl IP lysis (2.5 mM Tris, 150 mM NaCl, 1% Triton X-100, 1 mM EDTA, cocktail). Cells were lysed by vortexing vigorously and left on ice for 30 min. After centrifugation at 12,000 rpm for 30 min, the supernatant was transferred to a fresh tube and stored at -80°C. The total cellular lysates were then separated by 12% SDS-polyacrylamide gel electrophoresis (SDS-PAGE) and transferred onto polyvinylidene difluoride (PVDF) membranes. Membranes were blocked with 5% non-fat milk for 30 min at room temperature and incubated with primary antibodies at 1:200. Antibodies against Girdin, Cortactin, and actin (used as a loading control) were incubated overnight at 4°C, and HRP-conjugated secondary antibodies at 1:1000 dilution were incubated for 1 h at room temperature. The antigen-antibody complex was detected with the SuperEnhanced chemiluminescence (ECL) method according to the manufacturer’s recommendations. The relative quantities of proteins were determined by densitometer and expressed in absorbance units (AU). The cropped images for western blots are shown in the main figures. The experiments were repeated at least three times.

### CCK8 assay

Cell numbers were assayed with a cell counting kit-8 (CCK8). After PANC-1 and AsPC-1 cells were treated with either shGirdin (50 MOI) or shCtrl (50 MOI) for 24 h, were harvested and plated at 4000 cells per well with 100 μl RPMI-1640 medium in 96-well plate. After 48 h, 72 h, and 96 h incubation, 10μL CCK8 reaction solution was added into cultured medium in each well for 1 h at 37°C. Optical density was measured at 450 nm by a microplate reader.

### Apoptosis analysis by flow cytometry

Forty-eight hours after infecting the cells, the medium was replaced with fresh complete medium for another 48 h. Cells were harvested using trypsin free of EDTA, and collected in 1.5 ml Eppendorf tubes. Cells were washed with PBS twice. The pellet was re-suspended in 500 μl binding buffer, mixed gently. Cells were counted and adjusted to 2 × 10^6^ per ml. Cell suspension (100 μl) was transferred to test tubes, and 5 μl Annexin V-APC was added and mixed gently. Then, 5 μl propidium iodide (PI) was added and incubated for 10 min at room temperature avoid of light. 400 μl binding buffer was added to each tube and mixed. Samples were analyzed using a flow cytometer. Data analysis was performed using FlowJo software. All experiments were conducted three times.

### Wound-healing assay

The effect of Girdin on cell migration *in vitro* was analyzed with a wound-healing assay. Briefly, the outer bottom of 6-well culture plates was marked with a reference point for image acquisition. PANC-1 and AsPC-1 cells were plated to 70% confluence as a monolayer in the marked 6-well plates and treated with shGirdin (50 MOI), or shCtrl (50 MOI) in culture medium for 24 h. Scratches were then created in the monolayer with a pipette tip, and the wells were gently washed to remove the debris. RPMI1640 medium containing 2% fetal bovine serum was then added during to eliminate the effect of cell proliferation on cell migration. Progression of cell migration was observed and photographed under microscopy immediately after wounding and at 24 h after wounding. The gap distances were then quantitatively calculated using ImageJ software.

### Transwell invasion assay

The effect of Girdin on cell invasion *in vitro* was assessed with a transwell invasion assay. Briefly, 12.5μl Matrigel (50mg/l) was diluted in 87.5μl serum-free RPMI-1640 medium. The 100μl Matrigel-diluted solution was added to a 24-well transwell chamber, dried in a laminar hood for 20 min and reconstituted in 100μl serum-free RPMI1640 medium for 2 h. The 50 MOI shGirdin or shCtrl infected cells (2 × 10^4^ cells per 100μl serum-free RPMI-1640 medium) were added to the upper chamber. The lower chamber was filled with 600μl of culture medium. After 24 h of incubation, cells on the upper surface of filter were removed, and cells invading the bottom side of insert were fixed in absolute methanol for 10 min, stained with 600μl DAPI for 30 min, photographed, and quantified by counting five randomly selected 200X high-power fields of view. The invasive ability of tumor cells was then analyzed.

### In vivo xenograft experiments

Male BALB/c nude mice (6-week-old; n=6/group) were purchased from Beijing HFK Bioscience Co. Ltd. (Beijing, China) and maintained under pathogen-free conditions with approval by the Committee of the Second Affiliated Hospital of Nanjing Medical University. For tumor propagation analysis, 1×10^7^ PANC-1 tumor cells were subcutaneously injected into BALB/c nude mice. Tumor volume was calculated using the formula: volume = πab^2^/6 (a, tumor length; b, tumor width) at indicative time points. Tumor weight was measured at day 28 post-injection. Animal experiments were performed in accordance with relevant guidelines and regulations of the Animal Care and Use Committees at the Second Affiliated Hospital of Nanjing Medical University.

### Statistical analysis

Data from at least three independent experiments are expressed as the mean ± standard deviation. Statistical analysis was performed using IBM SPSS 19.0 statistical software. Statistical analysis was performed using Student’s t-test or ANOVA. Pearson’s χ2 tests were used for analysis of the correlation between clinicopathological features and Girdin expression in pancreatic cancer patients. P<0.05 was considered to indicate a statistically significant difference.

### Data availability statement

The data in the current study will be available from the corresponding author on a reasonable request.

## Supplementary Material

Supplementary Table 1

Supplementary Table 2

Supplementary File 1

Supplementary File 2

## References

[1] Bosetti C, Bertuccio P, Negri E, La Vecchia C, Zeegers MP, Boffetta P. Pancreatic cancer: overview of descriptive epidemiology. Mol Carcinog. 2012; 51:3–13. 10.1002/mc.20785 22162227

[2] Lillemoe KD. Current management of bile duct injury. Br J Surg. 2008; 95:403–05. 10.1002/bjs.6199 18320537

[3] Garcia G, Odaimi M. Systemic combination chemotherapy in elderly pancreatic cancer: A review. J Gastrointest Cancer. 2017; 48:121–28. 10.1007/s12029-017-9930-0 28303435

[4] Hampton T. New Target for Pancreatic Cancer Treatment Shows Potential. JAMA. 2019; 322:391–92. 10.1001/jama.2019.10165 31386111

[5] Wang X, Liu Q, Hou B, Zhang W, Yan M, Jia H, Li H, Yan D, Zheng F, Ding W, Yi C, Hai Wang. Concomitant targeting of multiple key transcription factors effectively disrupts cancer stem cells enriched in side population of human pancreatic cancer cells. PLoS One. 2013; 8:e73942. 10.1371/journal.pone.0073942 24040121 PMC3770686

[6] Zhan HX, Xu JW, Wu D, Zhang TP, Hu SY. Pancreatic cancer stem cells: new insight into a stubborn disease. Cancer Lett. 2015; 357:429–37. 10.1016/j.canlet.2014.12.004 25499079

[7] Fitzgerald TL, McCubrey JA. Pancreatic cancer stem cells: association with cell surface markers, prognosis, resistance, metastasis and treatment. Adv Biol Regul. 2014; 56:45–50. 10.1016/j.jbior.2014.05.001 24925031

[8] Lin L, Jou D, Wang Y, Ma H, Liu T, Fuchs J, Li PK, Lü J, Li C, Lin J. STAT3 as a potential therapeutic target in ALDH+ and CD44+/CD24+ stem cell-like pancreatic cancer cells. Int J Oncol. 2016; 49:2265–74. 10.3892/ijo.2016.3728 27748818 PMC5118001

[9] Kitamura T, Asai N, Enomoto A, Maeda K, Kato T, Ishida M, Jiang P, Watanabe T, Usukura J, Kondo T, Costantini F, Murohara T, Takahashi M. Regulation of VEGF-mediated angiogenesis by the Akt/PKB substrate Girdin. Nat Cell Biol. 2008; 10:329–37. 10.1038/ncb1695 18264090

[10] Ohara K, Enomoto A, Kato T, Hashimoto T, Isotani-Sakakibara M, Asai N, Ishida-Takagishi M, Weng L, Nakayama M, Watanabe T, Kato K, Kaibuchi K, Murakumo Y, et al. Involvement of Girdin in the determination of cell polarity during cell migration. PLoS One. 2012; 7:e36681. 10.1371/journal.pone.0036681 22574214 PMC3344933

[11] Ghosh P, Tie J, Muranyi A, Singh S, Brunhoeber P, Leith K, Bowermaster R, Liao Z, Zhu Y, LaFleur B, Tran B, Desai J, Jones I, et al. Girdin (GIV) Expression as a Prognostic Marker of Recurrence in Mismatch Repair-Proficient Stage II Colon Cancer. Clin Cancer Res. 2016; 22:3488–98. 10.1158/1078-0432.CCR-15-2290 27029492 PMC4947424

[12] Nishimae K, Tsunoda N, Yokoyama Y, Kokuryo T, Iwakoshi A, Takahashi M, Nagino M. The impact of Girdin expression on recurrence-free survival in patients with luminal-type breast cancer. Breast Cancer. 2015; 22:445–51. 10.1007/s12282-013-0501-3 24155038

[13] Cao K, Li J, Zhao Y, Wang Q, Zeng Q, He S, Yu L, Zhou J, Cao P. miR-101 inhibiting cell proliferation, migration and invasion in hepatocellular carcinoma through downregulating Girdin. Mol Cells. 2016; 39:96–102. 10.14348/molcells.2016.2161 26743900 PMC4757808

[14] Enomoto A, Murakami H, Asai N, Morone N, Watanabe T, Kawai K, Murakumo Y, Usukura J, Kaibuchi K, Takahashi M. Akt/PKB regulates actin organization and cell motility via Girdin/APE. Dev Cell. 2005; 9:389–402. 10.1016/j.devcel.2005.08.001 16139227

[15] Natsume A, Kato T, Kinjo S, Enomoto A, Toda H, Shimato S, Ohka F, Motomura K, Kondo Y, Miyata T, Takahashi M, Wakabayashi T. Girdin maintains the stemness of glioblastoma stem cells. Oncogene. 2012; 31:2715–24. 10.1038/onc.2011.466 22020337

[16] Pan S, Brentnall TA, Chen R. Proteomics analysis of bodily fluids in pancreatic cancer. Proteomics. 2015; 15:2705–15. 10.1002/pmic.201400476 25780901 PMC4526406

[17] Maryáš J, Faktor J, Dvořáková M, Struhárová I, Grell P, Bouchal P. Proteomics in investigation of cancer metastasis: functional and clinical consequences and methodological challenges. Proteomics. 2014; 14:426–40. 10.1002/pmic.201300264 24272997

[18] Silva AM, Vitorino R, Domingues MR, Spickett CM, Domingues P. Post-translational modifications and mass spectrometry detection. Free Radic Biol Med. 2013; 65:925–41. 10.1016/j.freeradbiomed.2013.08.184 24002012

[19] Thomas SN, Yang AJ. Mass Spectrometry Analysis of Lysine Posttranslational Modifications of Tau Protein from Alzheimer’s Disease Brain. Methods Mol Biol. 2017; 1523:161–177. 10.1007/978-1-4939-6598-4_10 27975250 PMC5220582

[20] Choi JS, Kim KH, Oh E, Shin YK, Seo J, Kim SH, Park S, Choi YL. Girdin protein expression is associated with poor prognosis in patients with invasive breast cancer. Pathology. 2017; 49:618–26. 10.1016/j.pathol.2017.05.010 28818465

[21] Wang S, Lei Y, Cai Z, Ye X, Li L, Luo X, Yu C. Girdin regulates the proliferation and apoptosis of pancreatic cancer cells via the PI3K/Akt signalling pathway. Oncol Rep. 2018; 40:599–608. 10.3892/or.2018.6469 29901184 PMC6072288

[22] Yang Z, Yang F, Zhang Y, Wang X, Shi J, Wei H, Sun F, Yu Y. Girdin protein: A potential metastasis predictor associated with prognosis in lung cancer. Exp Ther Med. 2018; 15:2837–43. 10.3892/etm.2018.5773 29456687 PMC5795640

[23] Wang H, Zhang J, Zhang M, Wei L, Chen H, Li Z. A systematic study of Girdin on cell proliferation, migration and angiogenesis in different breast cancer subtypes. Mol Med Rep. 2017; 16:3351–56. 10.3892/mmr.2017.6971 28713924

[24] Zhou Z, Chen Y, Jin M, He J, Guli A, Yan C, Ding S. Comprehensive analysis of lysine acetylome reveals a site-specific pattern in rapamycin-induced autophagy. J Proteome Res. 2019; 18:865–77. 10.1021/acs.jproteome.8b00533 30592415

[25] Jiang M, Hua Z, Dong Y, Liu Z, Thiele CJ, Li Z. Quantitative ubiquitylome analysis and crosstalk with proteome/acetylome analysis identified novel pathways and targets of perifosine treatment in neuroblastoma. Transl Cancer Res. 2018; 7:1548–60. 10.21037/tcr.2018.11.30 30761266 PMC6370305

[26] Hsu HC, You JF, Chen SJ, Chen HC, Yeh CY, Tsai WS, Hung HY, Yang TS, Lapke N, Tan KT. *TP53* DNA Binding Domain Mutations Predict Progression-Free Survival of Bevacizumab Therapy in Metastatic Colorectal Cancer. Cancers (Basel). 2019; 11:1079. 10.3390/cancers11081079 31366114 PMC6721375

[27] Iurescia S, Fioretti D, Rinaldi M. Targeting cytosolic nucleic acid-sensing pathways for cancer immunotherapies. Front Immunol. 2018; 9:711. 10.3389/fimmu.2018.00711 29686682 PMC5900005

[28] Guo B, Zhang J, Li Q, Zhao Z, Wang W, Zhou K, Wang X, Tong D, Zhao L, Yang J, Huang C. Hypermethylation of miR-338-3p and Impact of its Suppression on Cell Metastasis Through N-Cadherin Accumulation at the Cell -Cell Junction and Degradation of MMP in Gastric Cancer. Cell Physiol Biochem. 2018; 50:411–25. 10.1159/000494153 30308487

[29] Wiese EK, Hitosugi T. Tyrosine kinase signaling in cancer metabolism: PKM2 paradox in the warburg effect. Front Cell Dev Biol. 2018; 6:79. 10.3389/fcell.2018.00079 30087897 PMC6066570

[30] Xie S, Liu Y, Li X, Tan M, Wang C, Field J, Zhou GL. Phosphorylation of the Cytoskeletal Protein CAP1 Regulates Non-Small Cell Lung Cancer Survival and Proliferation by GSK3β. J Cancer. 2018; 9:2825–33. 10.7150/jca.25993 30123351 PMC6096378

[31] Cai G, Wu D, Wang Z, Xu Z, Wong KB, Ng CF, Chan FL, Yu S. Collapsin response mediator protein-1 (CRMP1) acts as an invasion and metastasis suppressor of prostate cancer via its suppression of epithelial-mesenchymal transition and remodeling of actin cytoskeleton organization. Oncogene. 2017; 36:546–58. 10.1038/onc.2016.227 27321179 PMC5290039

[32] Zhang X, Liu K, Zhang T, Wang Z, Qin X, Jing X, Wu H, Ji X, He Y, Zhao R. Cortactin promotes colorectal cancer cell proliferation by activating the EGFR-MAPK pathway. Oncotarget. 2017; 8:1541–54. 10.18632/oncotarget.13652 27903975 PMC5352075

[33] MacGrath SM, Koleske AJ. Cortactin in cell migration and cancer at a glance. J Cell Sci. 2012; 125:1621–26. 10.1242/jcs.093781 22566665 PMC3346822

