# Supplementary material for: Quantitative acetylome and phosphorylome analysis reveals Girdin affects pancreatic cancer progression through regulating Cortactin
Source: Aging (Albany NY). 2020 May 5;12(9):7679–93. doi: 10.18632/aging.103032 (PMC7244020; doi:10.18632/aging.103032)
Supplement: Supplementary File 2 [file aging-12-103032-s003..pdf]

Proteomic Quantification of Phosphorylation in *Human Cell Line*

(Project Report)

Project No.: **5144SP02**

Prepared for: 南京医科大学第二附属医院

Start Date: **10/10/2015**

Report Date: **11/09/2015**

CONTACT INFORMATION:

Wenkai Qiu, Sales Representative

Mobile: + 186 5880 9390

Email: wenkai_qiu@ptm-biolab.com

PTM-Biolabs (HangZhou) Co., Ltd

No.452, 6th Street, No. 2, 15 floor Area A, Hangzhou Eco & Tech Developmental Area

Hangzhou, Zhejiang, China, 310018

Table of Contents

Summary 4

Technical Route 5

1. Results 6

1.1. Quantitative Overview 6

1.2. Protein Annotation 6

1.3. Motif Analysis 7

1.4. Functional Classification of Differentially Quantified Proteins 8

1.4.1. GO Classification of Terms Level 2 8

1.4.2. Subcellular Location Classification 10

1.5. Functional Enrichment of Differentially Quantified Proteins 12

1.5.1. GO Enrichment 12

1.5.2. KEGG Pathway Enrichment 13

1.5.3. Domain Enrichment 14

1.5.4. Complex Enrichment 15

1.6. Cluster Analysis 16

1.6.1. Quantiles-based Clustering for Protein Groups 16

2. Suggestions for Further Studies 18

3. Materials and Methods 18

3.1. Sample Preparation 18

3.1.1. Materials and Reagents 18

3.1.2. SILAC Labeling 19

3.1.3. Protein Extraction 20

3.1.4. Trypsin Digestion 20

3.1.5. HPLC Fractionation 20

3.1.6. Affinity Enrichment 20

3.2. Quantitative Proteomic Analysis by LC-MS/MS 21

3.2.1. Materials and Reagents 21

3.2.2. Mass Spectrometer 21

3.2.3. LC-MS/MS Analysis 21

3.2.4. Database Search 22

3.2.5. QC Validation of MS Data 22

3.3. Bioinformatics Methods 24

3.3.1. Annotation Methods 24

3.3.2. Motif Analysis 27

3.3.3. Functional Enrichment 27

3.3.4. Enrichment-based Clustering 28

Summary

Using SILAC labeling and affinity enrichment followed by high-resolution LC-MS/MS analysis, quantitative phosphorylome analysis was performed in pair of *human cell lines*. Altogether, **5,468** phosphorylation sites in **2,317** protein groups were identified, among which **5,338** sites in **2,263** proteins were quantified. When setting quantification ratio of >1.5 as up-regulated threshold and <0.67 as down-regulated threshold, **657** phosphorylation sites in **474** proteins were quantified as up-regulated targets and **404** phosphorylation sites in **307** proteins were quantified as down-regulated targets. Intensive bioinformatic analysis was then carried out to annotate those quantifiable targets, including protein annotation, functional classification, functional enrichment, functional enrichment-based cluster analysis, *etc*. Based on the results, further studies following the quantitative proteome analysis were suggested.

Technical Route

The aim of this project is to use integrated approach involving SILAC labeling, HPLC fractionation, affinity enrichment, mass spectrometry-based quantitative proteomics to quantify dynamic changes of the whole phosphorylome of *human cell lines*. The general technical route is indicated below:

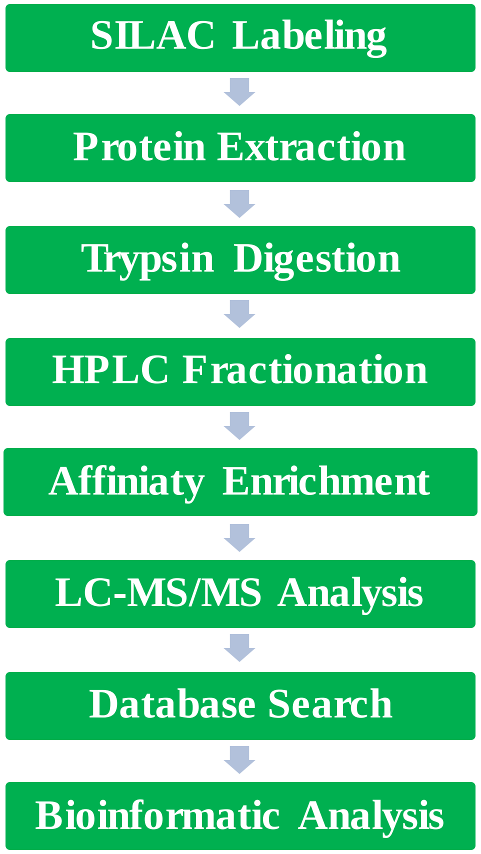

PTM-Biolabs (HangZhou) Co., Ltd
No.452, 6th Street, No. 2, 15 floor Area A, Hangzhou Eco & Tech Developmental Area,
Hangzhou, Zhejiang, China, 310018

1. Results

1.1. Quantitative Overview

Altogether, **5,468** phosphorylation sites in **2,317** proteins were identified, among which **5,338** phosphorylation site in **2,263** proteins were quantified (**Table 1**). All the data was presented in the file of “5144SP_Pho/2-Basic_analysis/ SILAC_modified_quantification.xlsx”

In this report, the quantitative ratio over 1.5 was considered as up-regulation while quantitative ratio below 1/1.5 (0.67) was considered as down-regulation (T test p-value <0.05). The number of differentially quantified sites and proteins were summarized in **Table 2.**

Table 1: Summary of identified and quantified sites and proteins

Name	Identified	Quantified
**Sites**	5,468	5,338
**Proteins**	2,317	2,263

Table 2: Summary of differentially quantified sites and proteins (>1.5, or <0.67)

Name	Up-regulated (>1.5)	Down-regulated (<0.67)
**Sites**	657	404
**Proteins**	474	307

1.2. Protein Annotation

To further understand the function and feature of identified and quantified proteins, we annotated function or feature of protein from several different categories, including Gene Ontology, Protein Domain, Protein Complex, KEGG Pathway and Subcellular Localization. Firstly, all the identified proteins were annotated. Then, the quantifiable proteins were also annotated.

The results were presented in the folder: 5144SP_Pho/4-Protein_annotation

1.3. Motif Analysis

Motif Logo	Motif	Motif Score	Foreground		Background		Fold Increase
			Matches	Size	Matches	Size	
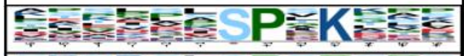	......SP.K....	32	215	4763	862	183075	9.59
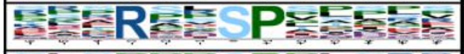	...R..SP.....	25.96	199	4548	1027	182213	7.76
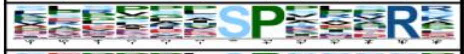	......SP...R.	25.31	161	4349	838	181186	8
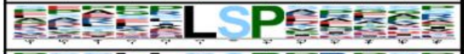	.....LSP.....	24.87	200	4188	1177	180348	7.32
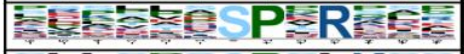	......SP.R....	23.33	124	3988	717	179171	7.77
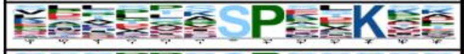	......SP..K..	23.36	99	3864	558	178454	8.19
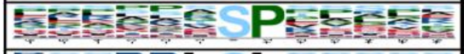	......SP.....	16	947	3765	9905	177896	4.52
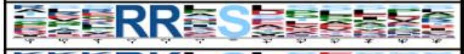	...RR.S......	32	228	2818	1017	167991	13.36
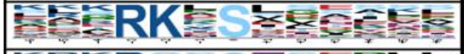	...RK.S......	32	122	2590	729	166974	10.79
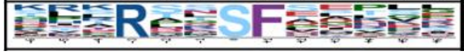	...R..SF.....	26.36	54	2468	329	166245	11.06
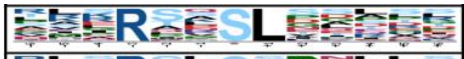	...R..SL.....	27.41	107	2414	964	165916	7.63
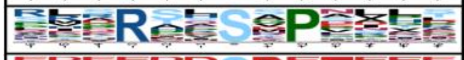	...R..S.P....	25.77	62	2307	526	164952	8.43
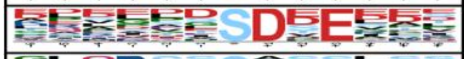	......SD.E....	32	92	2245	1154	164426	5.84
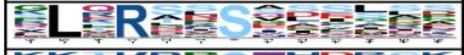	.L.R..S......	24.44	70	2153	768	163272	6.91
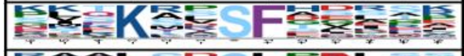	...K..SF.....	30.05	42	2083	377	162504	8.69
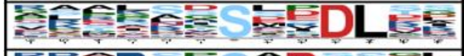	......S..DL..	23.06	54	2041	904	162127	4.75
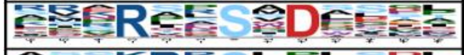	...R..S.D....	22.14	36	1987	386	161223	7.57
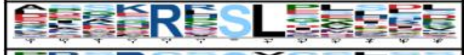	....R.SL.....	25.43	57	1951	791	160837	5.94
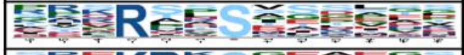	...R..S......	16	211	1894	6099	160046	2.92
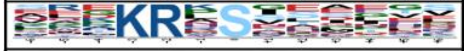	...KR.S......	22.6	33	1683	561	153947	5.38
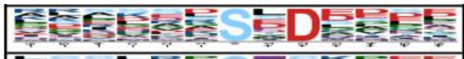	......S.D....	16	196	1650	8066	153386	2.26
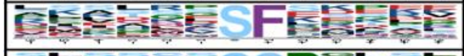	......SF.....	14.7	106	1454	4500	145320	2.35
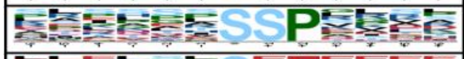	.....SSP.....	31.95	82	1348	1839	140820	4.66
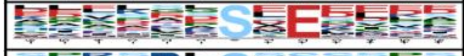	......S.E....	13.92	176	1266	10557	138981	1.83
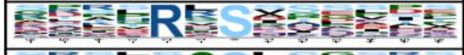	....R.S......	12.75	102	1090	5424	128424	2.22
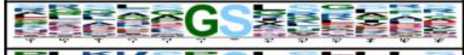	.....GS......	13.75	135	988	8363	123000	2.01
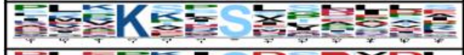	...K..S......	14.57	119	853	7353	114637	2.17
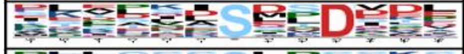	......S..D...	9.1	75	734	5097	107284	2.15
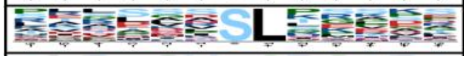	......SL.....	9.26	122	659	10733	102187	1.76

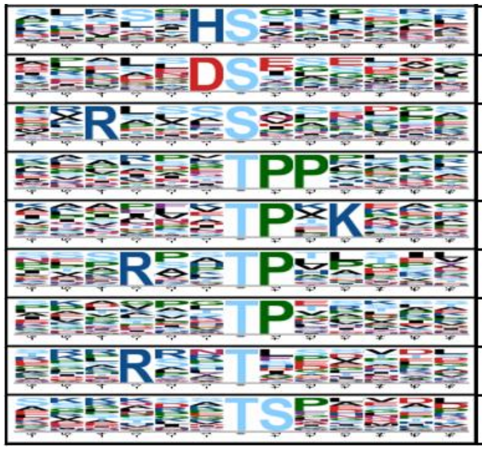

......HS......	8.31	42	537	2571	91454	2.78
......DS......	6.9	60	495	5224	88883	2.06
..R...S......	6.37	48	435	4213	83659	2.19
......TPP....	32	83	549	820	105492	19.45
......TP.K...	24.16	39	466	470	104672	18.64
...R..TP.....	23.01	31	427	405	104202	18.68
......TP.....	16	161	396	6892	103797	6.12
...R..T......	16	53	235	4917	96905	4.44
......TS.....	8.93	49	182	9934	91988	2.49

Figure 1. Motif analysis of all the identified phosphorylation sites.

The results were presented in the folder: 5144SP_Pho/9-Motif_analysis

1.4. Functional Classification of Differentially Quantified Proteins

1.4.1. GO Classification of Terms Level 2

According to GO annotation information of identified phosphorylation proteins, we calculated the number of quantifiable proteins in each GO term of level 2.

Table 3: The GO terms of level 2 distribution of up-regulated phosphorylation proteins (L/H)

GO Terms Level 1	GO Terms Level 2	No. of Protein
**Biological Process**	cellular process	382
	single-organism process	343
	biological regulation	315
	metabolic process	236
	response to stimulus	209
	cellular component organization or biogenesis	180
	multicellular organismal process	173
	developmental process	161
	signaling	155
	localization	125
	multi-organism process	59
	other	165
**Cellular Component**	cell	422
	organelle	359
	macromolecular complex	174

	membrane	169
	membrane-enclosed lumen	134
	cell junction	51
	other	33
**Molecular Function**	binding	311
	catalytic activity	136
	enzyme regulator activity	51
	nucleic acid binding transcription factor activity	35
	protein binding transcription factor activity	30
	molecular transducer activity	22
	structural molecule activity	16
	other	26

Table 4: The GO terms of level 2 distribution of down-regulated phosphorylation proteins (L/H)

GO Terms Level 1	GO Terms Level 2	No. of Protein
**Biological Process**	cellular process	240
	single-organism process	230
	biological regulation	202
	response to stimulus	139
	metabolic process	123
	cellular component organization or biogenesis	113
	multicellular organismal process	111
	developmental process	107
	signaling	100
	localization	82
	immune system process	33
	multi-organism process	32
	other	76
**Cellular Component**	cell	282
	organelle	221
	membrane	134
	macromolecular complex	101
	membrane-enclosed lumen	62
	cell junction	30
	extracellular region	18
	other	14
**Molecular Function**	binding	197
	catalytic activity	97
	enzyme regulator activity	30

structural molecule activity	20
protein binding transcription factor activity	18
molecular transducer activity	14
transporter activity	13
receptor activity	10
other	7

Note: For detailed information, please find the corresponding excel files in the supplementary folder of "5144SP_Pho/5-Functional_classification".

1.4.2. Subcellular Location Classification

According to subcellular location annotation information of identified phosphorylation proteins, we calculated the number of quantifiable proteins in each subcellular location.

Table 5: The subcellular location of up-regulated phosphorylation proteins (L/H)

Subcellular Location	No. of Protein
nucl	312
plas	44
cyto	66
mito	23
cyto_nucl	13
extr	6
golg	1
cysk	3
cyto_mito	1
E.R.	2
extr_plas	2
E.R._mito	1

Table 6: The subcellular location of down-regulated phosphorylation proteins (L/H)

Subcellular Location	No. of Protein
nucl	177
cyto	54
extr_plas	3
plas	28
mito	22
cyto_nucl	9
E.R.	3
extr	9

pero	1
cysk	1

Note: For detailed information, please find the corresponding excel files in the supplementary folder of "5144SP_Pho/5-Functional_classification".

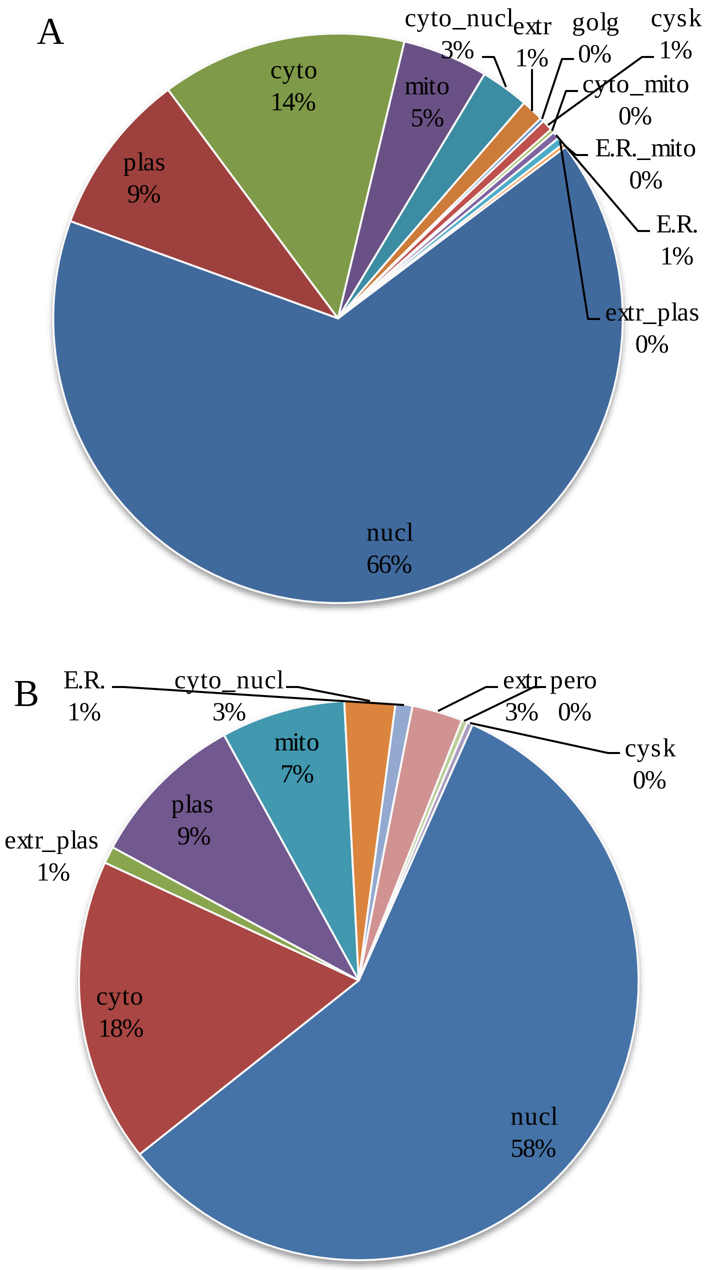

Figure 2. The subcellular location of up-regulated (A) and down-regulated (B) proteins (L vs H). For detained information of the subcellular location of up- and down-regulated proteins, please

find the corresponding excel files in the supplementary folder of "5144SP_Pho/5-Functional_classification".

The results were presented in the folder: 5144SP_Pho/5-Functional_classification

1.5. Functional Enrichment of Differentially Quantified Proteins

1.5.1. GO Enrichment

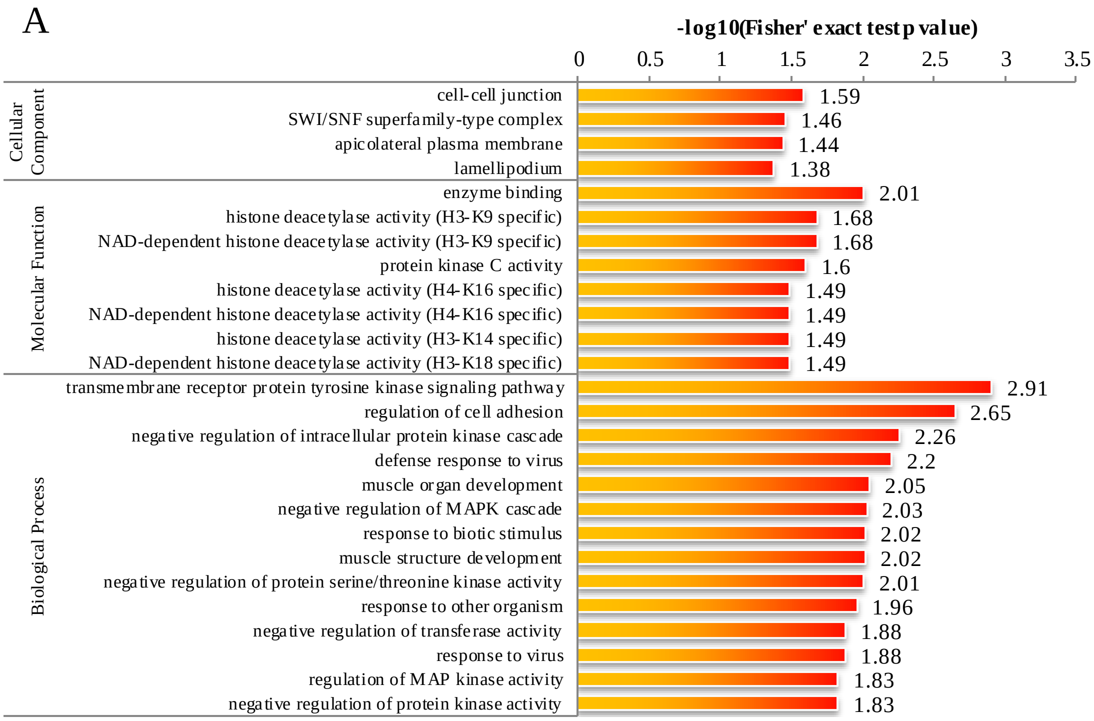

B

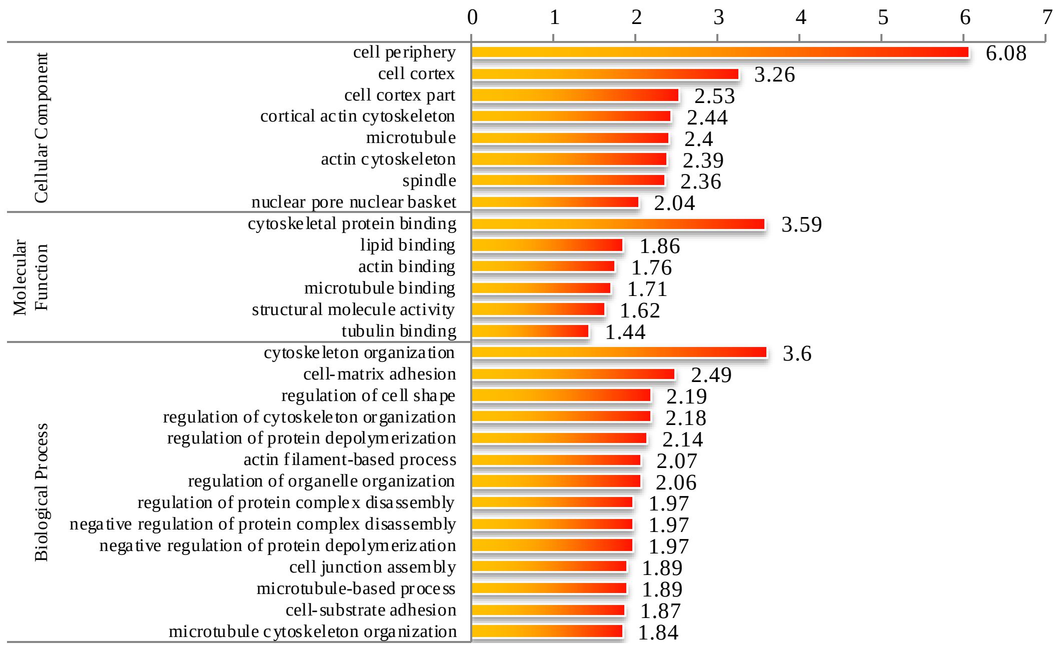

Figure 3. GO-based enrichment analysis of up-regulated (A) and down-regulated (B) proteins (L vs H). For detailed information of GO-based enrichment, please find the corresponding excel files in supplementary folder of “5144SP_Pho/6-Functional_enrichment”.

1.5.2. KEGG Pathway Enrichment

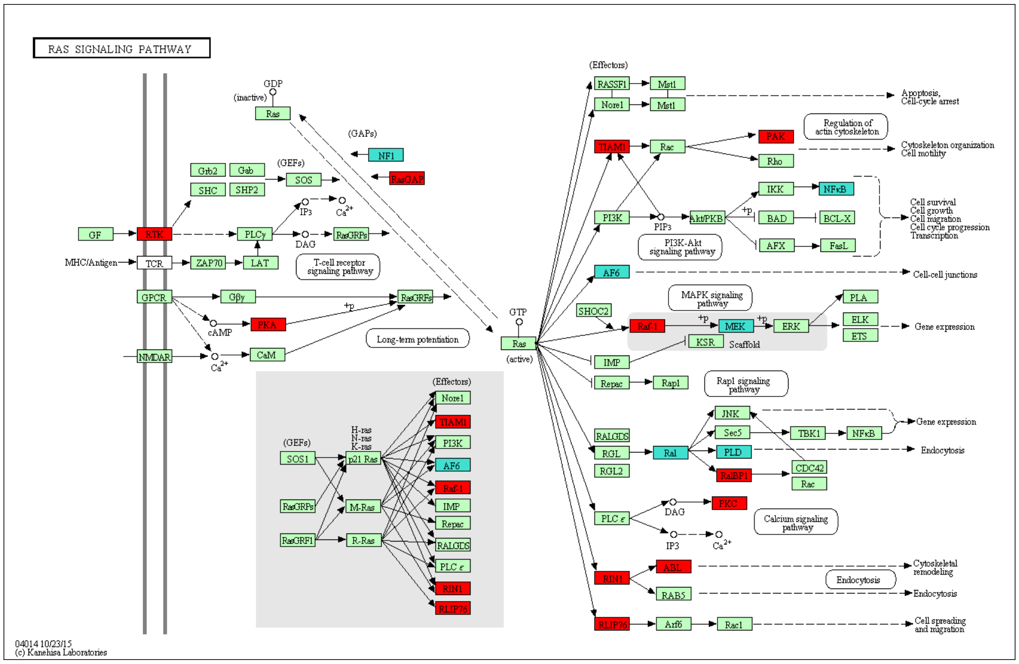

Figure 4. The pathway obtained from KEGG pathway enrichment analysis (L vs H). The proteins in turquoise are down-regulated and the proteins in red are up-regulated. For detailed information of KEGG pathway, please find the supplementary folder of “5144SP_Pho/8-Enrichment_pathway_image”.

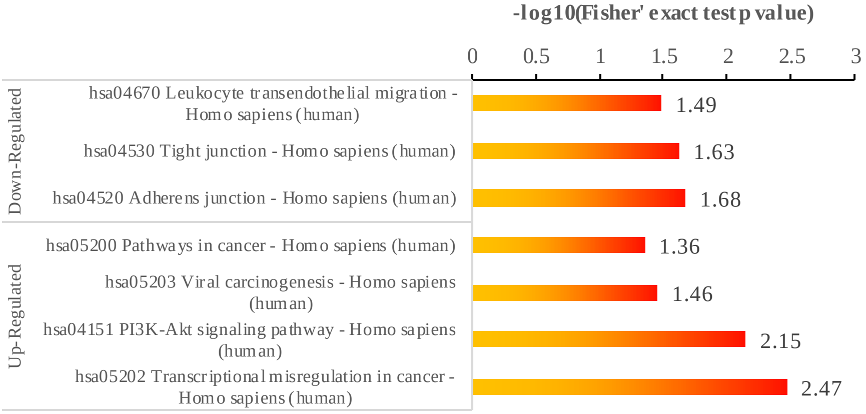

Figure 5. KEGG pathway-based enrichment analysis of up- and down-regulated proteins (L vs H). For detailed information of KEGG pathway, please find the corresponding excel files in supplementary folder of “5144SP_Pho/6-Functional_enrichment”.

1.5.3. Domain Enrichment

A

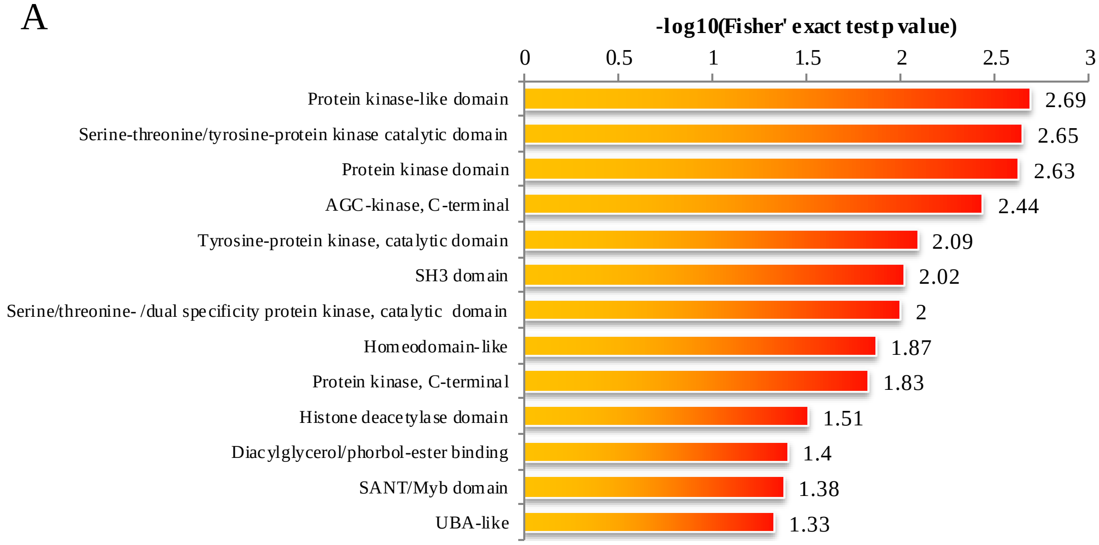

B

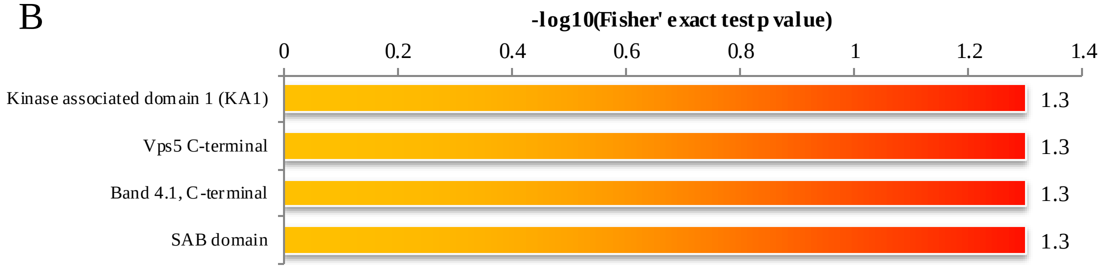

Figure 6. Protein domain enrichment analysis of up-regulated (A) and down-regulated (B) proteins (L vs H). For detailed information of protein domain, please find the corresponding excel files in supplementary folder of “5144SP_Pho/6-Functional_enrichment”.

1.5.4. Complex Enrichment

A

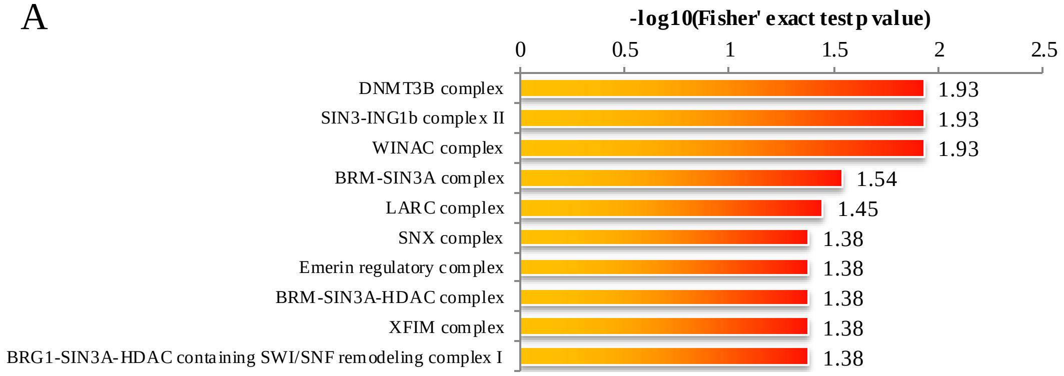

B

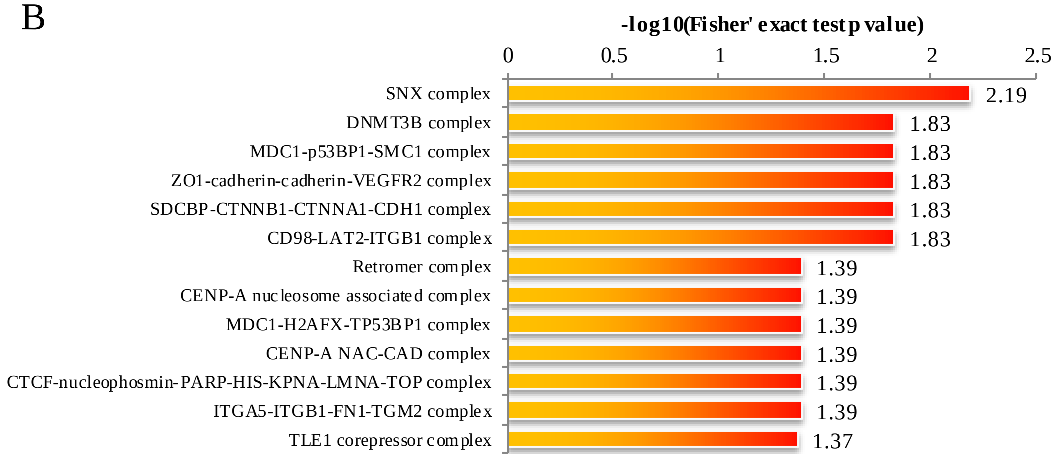

Figure 7. Protein complex enrichment analysis of up-regulated (A) and down-regulated (B) proteins (L vs H). For detailed information of protein complex, please find the corresponding excel files in supplementary folder of “5144SP_Pho/6-Functional_enrichment”.

The results were presented in the folder: 5144SP_Pho/6-Functional_enrichment

1.6. Cluster Analysis

1.6.1. Quantiles-based Clustering for Protein Groups

Firstly, the quantified proteins in this study were divided into four quantiles according to the quantification ratio to generated four quantiles: Q1 (0~1/1.5), Q2 (1/1.5~1/1.3), Q3 (1.3~1.5) and Q4 (>1.5). Then, the quantiles-based clustering was performed.

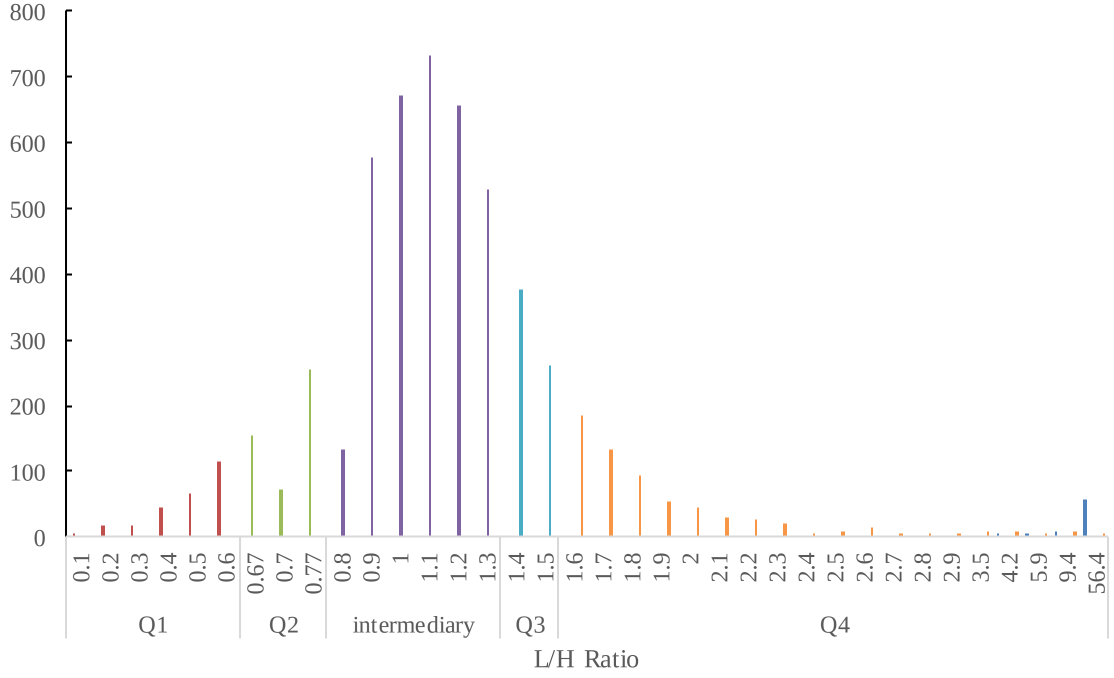

Figure 8. Distribution of quantification results.

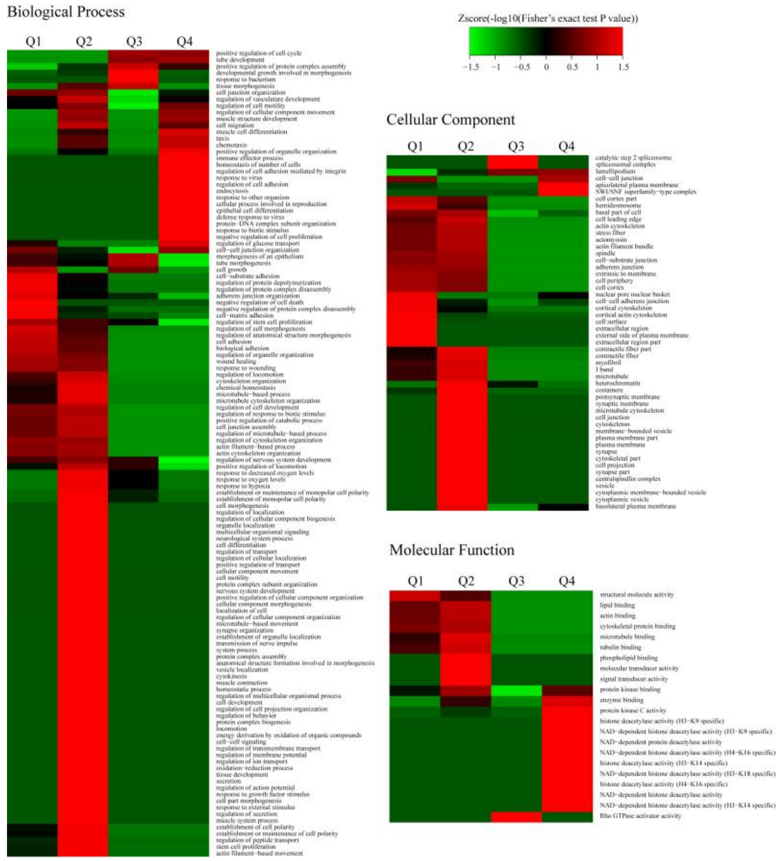

Figure 9. GO enrichment-based cluster analysis.

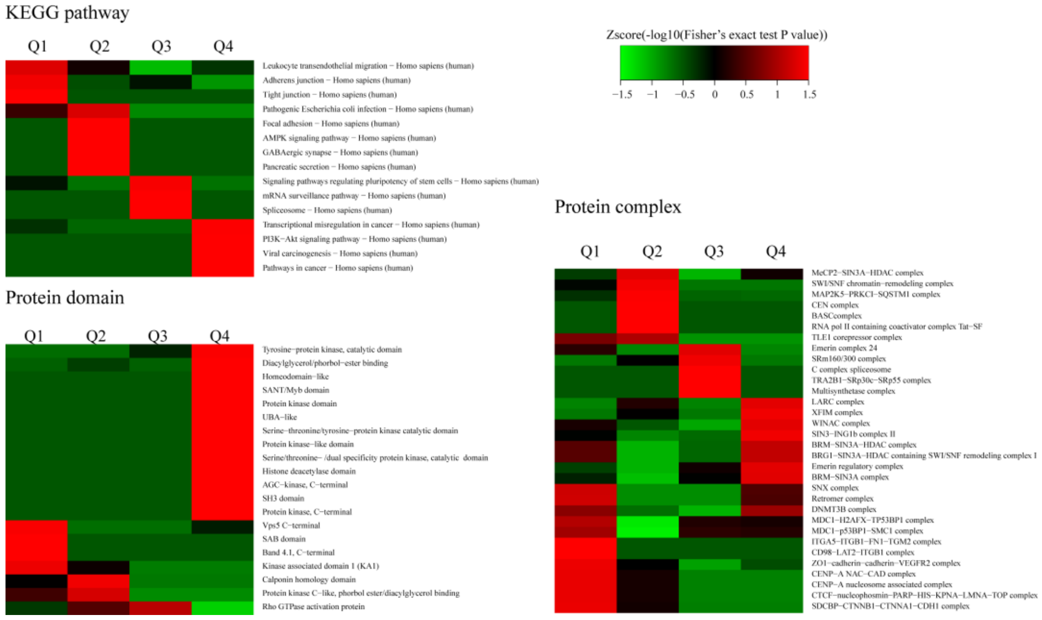

Figure 10. Protein domain, Protein complex and KEGG pathway-based clustering analysis.

The results were presented in the folder: 5144SP_Pho/7-Functional_enrichment_cluster

2. Suggestions for Further Studies

Based on the results of the current project, the following studies are recommended by PTM Biolabs:

- **To investigate the crosstalk between ubiquitination and phosphorylation**

Ubiquitination and phosphorylation are well known to have intensive crosstalk with vital biological functions. PTM Biolabs has reagents and stable technologies for the quantification of both ubiquitination and phosphorylation. The bioinformatic analysis of the crosstalk between ubiquitination and phosphorylation could also be deeply performed by the advanced bioinformatics team in PTM Biolabs.

- **To investigate other PTMs and their crosstalks**

PTM Biolabs also has reagents and technologies for determining dynamic changes of other PTM substrates and the crosstalk between them, using in-house established methodologies, including Phosphorylation (phosphorylation), lysine succinylation (Ksucc), lysine glutarylation (Kglu), lysine butyrylation (Kbuty), lysine propionylation (Kprop), lysine crotonylation (Kcr), O-GlcNAcylatio, N-glycosylation and phosphoryaltion.

PTM Biolabs is able to deliver high quality service in the projects proposed above. We are proudly providing our technical expertise and knowledge in epigenetics and proteomics towards the client’s success.

3. Materials and Methods

3.1. Sample Preparation

3.1.1. Materials and Reagents

Name	Company	CAT#.
DMEM Medium	Pierce	DMEM Medium
DMEM-L	Gibco	DMEM-L

FBS	Gibco	FBS
SILAC™ Protein Identification and Quantitation Media Kit	Thermo	SILAC™ Protein Identification and Quantitation Media Kit
Sequencing Grade Modified Trypsin	Promega	V5111
ACN (acetonitrile)	Fisher	A998-4
TFA (trifluoroacetic acid)	Sigma	302031-100ML
FA (formic acid)	Fluka	56302-50ML-F
IAA (iodoacetamide)	Sigma	
DTT (dithiothreitol)	Sigma	
2-D Quant kit	GE Healthcare	

3.1.2. SILAC Labeling

The cells were grown to 80% confluence in high glucose (4.5 g/liter) Dulbecco's modified Eagle's medium (with glutamine and sodium pyruvate) containing 10% fetal bovine serum and 1% penicillin-streptomycin at 37 ℃ with 95% air and 5% CO_2. The cells were labeled with either "heavy isotopic lysine" (^{13}C-Lysine) or "light isotopic lysine" (^{12}C-Lysine) using a SILAC Protein Quantitation Kit (Pierce, Thermo) according to manufacturer's instructions. Briefly, the cell line was grown in Dulbecco's modified Eagle's medium supplemented with 10% fetal bovine serum and either the "heavy" form of [U-$^{13}C_6$]- L-lysine or "light" [U-$^{12}C_6$]- L-lysine for more than six generations before being harvested, to achieve more than 97% labeling efficiency. After that, the cells were further expanded in SILAC media to desired cell number (~5×10^8) in fifteen 150 cm^2 flasks.

The "light" labeled cells were then treated with 5×10^{10} PFU/mL ad-girdin shRNA and the "heavy" labeled cells were treated with same amount of ad-GFP. After treatment, the cells were maintained in SILAC media for another 48 hours. The cells were then harvested and washed twice with ice-cold PBS. After snap freezing in liquid nitrogen, cell pellets were stored in -80 ℃ freezer for future use.

Table 2. Labeling information

Groups	Labeling information
ad-GFP	Heavy (lysine $^{13}C_6$ and arginine $^{13}C_6$,$^{15}N_4$)

ad-girdin shRNA Treated	Light

3.1.3. Protein Extraction

The harvested "heavy" and "light" labeled cells were lysed with lysis buffer (100 mM Tris-Cl, 2mM EDTA, pH 7.2) supplemented with Phosphatase Inhibitor Cocktail Set III and Protease Inhibitor Cocktail Set V on ice using a high intensity ultrasonic processor (Scientz) for 30 min, respectively. The supernatants were saved after centrifuge at 20,000 g for 10 min at 4 ℃. The protein concentration was determined with 2-D Quant kit according to the manufacturer's instructions.

3.1.4. Trypsin Digestion

For digestion, the protein solution was reduced with 10 mM DTT for 1 h at 37 ℃ and alkylated with 20 mM IAA for 45 min at room temperature in darkness. Finally, trypsin was added at 1:50 trypsin-to-protein mass ratio for the first digestion overnight and 1:100 trypsin-to-protein mass ratio for a second 4 h-digestion.

3.1.5. HPLC Fractionation

The sample was then fractionated into fractions by high pH reverse-phase HPLC using Agilent 300Extend C18 column (5 µm particles, 4.6 mm ID, 250 mm length). Briefly, peptides were first separated with a gradient of 2% to 60% acetonitrile in 10 mM ammonium bicarbonate pH 10 over 80 min into 80 fractions, Then, the peptides were combined into 8 fractions and dried by vacuum centrifuging.

3.1.6. Affinity Enrichment

Peptide mixtures were first incubated with IMAC microspheres suspension with vibration. The IMAC microspheres with enriched phosphopeptides were collected by centrifugation, and the supernatant was removed. To remove nonspecifically adsorbed peptides, the IMAC microspheres were washed with 50% ACN/6% TFA and 30% ACN/0.1% TFA, sequentially. To elute the enriched phosphopeptides from the IMAC microspheres, elution buffer containing 10% NH4OH

was added and the enriched phosphopeptides were eluted with vibration. The supernatant containing phosphopeptides was collected and lyophilized for LC-MS/MS analysis.

3.2. Quantitative Proteomic Analysis by LC-MS/MS

3.2.1. Materials and Reagents

Name	Company
H_2O	Thermo
ACN (acetonitrile)	Fisher Chemical
FA (formic acid)	Fluka

3.2.2. Mass Spectrometer

Thermo Scientific™ Q Exactive™ Plus

3.2.3. LC-MS/MS Analysis

Peptides were dissolved in solvent A (0.1% FA in 2% ACN), directly loaded onto a reversed-phase pre-column (Acclaim PepMap 100, Thermo Scientific). Peptide separation was performed using a reversed-phase analytical column (Acclaim PepMap RSLC, Thermo Scientific) with a linear gradient of 2-24% solvent B (0.1% FA in 98% ACN) for 50 min, 24–36% solvent B for 12 min and 35–80% solvent B for 4 min then holding at 80% for the last 4min, all at a constant flow rate of 300 nl/min on an EASY-nLC 1000 UPLC system. The resulting peptides were analyzed by Q Exactive™ Plus hybrid quadrupole-Orbitrap mass spectrometer (ThermoFisher Scientific).

The peptides were subjected to NSI source followed by tandem mass spectrometry (MS/MS) in Q Exactive™ Plus (Thermo) coupled online to the UPLC. Intact peptides were detected in the Orbitrap at a resolution of 70,000. Peptides were selected for MS/MS using NCE setting as 28; ion fragments were detected in the Orbitrap at a resolution of 17,500. A data-dependent procedure that alternated between one MS scan followed by 20 MS/MS scans was applied for the top 20 precursor ions above a threshold ion count of 5E3 in the MS survey scan with 15.0s dynamic exclusion. The electrospray voltage applied was 2.0 kV. Automatic gain control (AGC)

was used to prevent overfilling of the ion trap; 5E4 ions were accumulated for generation of MS/MS spectra. For MS scans, the m/z scan range was 350 to 1800.

3.2.4. Database Search

The resulting MS/MS data was processed using MaxQuant with integrated Andromeda search engine (v.1.4.1.2). Tandem mass spectra were searched against *SwissProt_Human (20,203 sequences)* database concatenated with reverse decoy database. Trypsin/P was specified as cleavage enzyme allowing up to 2 missing cleavages, 5 modifications per peptide and 5 charges. Mass error was set to 10 ppm for precursor ions and 0.02 Da for fragment ions. Carbamidomethylation on Cys was specified as fixed modification and oxidation on Met, phosphorylation on Ser, Thr, Tyr and acetylation on protein N-terminal were specified as variable modifications. False discovery rate (FDR) thresholds for protein, peptide and modification site were specified at 1%. Minimum peptide length was set at 7. All the other parameters in MaxQuant were set to default values. The site localization probability was set as > 0.75.

3.2.5. QC Validation of MS Data

The MS data validation was shown in **Figure 11**. Firstly, we checked the mass error of all the identified peptides. The distribution of mass error is near zero and most of them are less than 0.02 Da which means the mass accuracy of the MS data fit the requirement (**Figure 11A**). Secondly, the length of most peptides distributed between 8 and 20, which agree with the property of tryptic peptides (**Figure 11B**), that means sample preparation reach the standard.

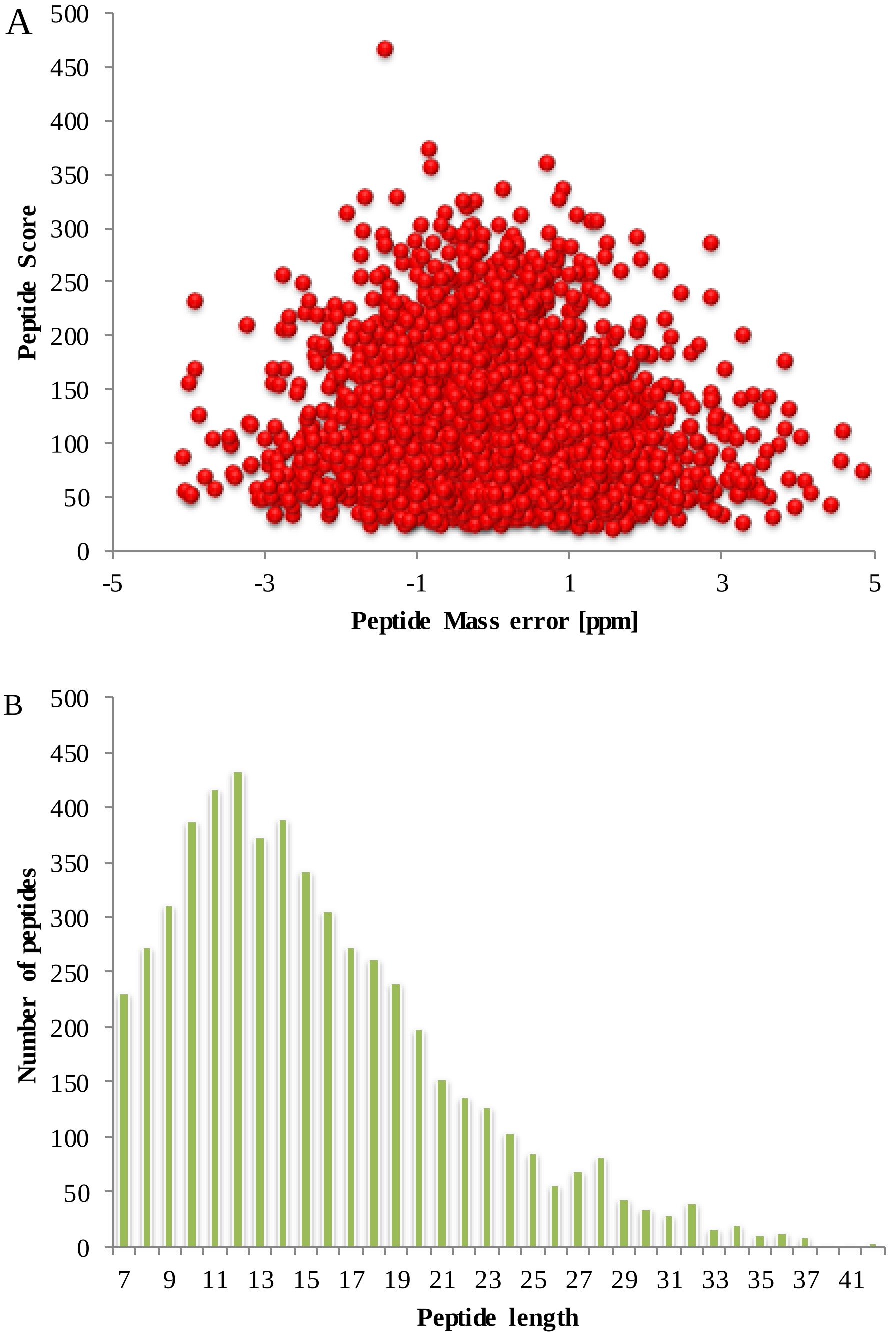

Figure 11. QC validation of MS data. (A) Mass error distribution of all identified peptides, (B) Peptide length distribution.

PTM-Biolabs (HangZhou) Co., Ltd
No.452, 6th Street, No. 2, 15 floor Area A, Hangzhou Eco & Tech Developmental Area, Hangzhou, Zhejiang, China, 310018

3.3. Bioinformatics Methods

3.3.1. Annotation Methods

GO Annotation

The Gene Ontology, or GO, is a major bioinformatics initiative to unify the representation of gene and gene product attributes across all species. More specifically, the project aims to:

1. Maintain and develop its controlled vocabulary of gene and gene product attributes;
2. Annotate genes and gene products, and assimilate and disseminate annotation data;
3. Provide tools for easy access to all aspects of the data provided by the project.

The ontology covers three domains:

1. Cellular component: A cellular component is just that, a component of a cell, but with the proviso that it is part of some larger object; this may be an anatomical structure (e.g. rough endoplasmic reticulum or nucleus) or a gene product group (e.g. ribosome, proteasome or a protein dimer).
2. Molecular function: Molecular function describes activities, such as catalytic or binding activities, that occur at the molecular level. GO molecular function terms represent activities rather than the entities (molecules or complexes) that perform the actions, and do not specify where or when, or in what context, the action takes place.
3. Biological process: A biological process is series of events accomplished by one or more ordered assemblies of molecular functions. It can be difficult to distinguish between a biological process and a molecular function, but the general rule is that a process must have more than one distinct steps.

Gene Ontology (GO) annotation proteome was derived from the UniProt-GOA database (www. http://www.ebi.ac.uk/GOA/). Firstly, Converting identified protein ID to UniProt ID and then mapping to GO IDs by protein ID. If some identified proteins were not annotated by UniProt-GOA database, the InterProScan soft would be used to annotated protein's GO functional based on protein sequence alignment method. Then proteins were classified by Gene Ontology annotationbased on three categories: biological process, cellular component and molecular function.

Domain Annotation

A protein domain is a conserved part of a given protein sequence and structure that can evolve, function and exist independently of the rest of the protein chain. Each domain forms a compact three-dimensional structure and often can be independently stable and folded. Many proteins consist of several structural domains. One domain may appear in a variety of different proteins. Molecular evolution uses domains as building blocks and these may be recombined in different arrangements to create proteins with different functions. Domains vary in length from between about 25 amino acids up to 500 amino acids in length. The shortest domains such as zinc fingers are stabilized by metal ions or disulfide bridges. Domains often form functional units, such as the calcium-binding EF hand domain of calmodulin. Because they are independently stable, domains can be "swapped" by genetic engineering between one protein and another to make chimeric proteins.

Identified proteins domain functional description were annotated by InterProScan (a sequence analysis application) based on protein sequence alignment method, and the InterPro domain database was used. InterPro (http://www.ebi.ac.uk/interpro/) is a database that integrates diverse information about protein families, domains and functional sites, and makes it freely available to the public via Web-based interfaces and services. Central to the database are diagnostic models, known as signatures, against which protein sequences can be searched to determine their potential function. InterPro has utility in the large-scale analysis of whole genomes and meta-genomes, as well as in characterizing individual protein sequences.

KEGG Pathway Annotation

KEGG connects known information on molecular interaction networks, such as pathways and complexes (the "Pathway" database), information about genes and proteins generated by genome projects (including the gene database) and information about biochemical compounds and reactions (including compound and reaction databases). These databases are different networks, known as the "protein network", and the "chemical universe" respectively. There are efforts in progress to add to the knowledge of KEGG, including information regarding ortholog clusters in the KEGG Orthology database. KEGG Pathways mainly including: Metabolism, Genetic Information Processing, Environmental Information Processing, Cellular Processes, Rat Diseases, Drug development. Kyoto Encyclopedia of Genes and Genomes (KEGG)database was used to annotate protein pathway. Firstly, using KEGG online service tools KAAS to annotated protein's

KEGG database description. Then mapping the annotation result on the KEGG pathway database using KEGG online service tools KEGG mapper.

Subcellular Localization

The cells of eukaryotic organisms are elaborately subdivided into functionally distinct membrane bound compartments. Some major constituents of eukaryotic cells are: extracellular space, cytoplasm, nucleus, mitochondria, Golgi apparatus, endoplasmic reticulum (ER), peroxisome, vacuoles, cytoskeleton, nucleoplasm, nucleolus, nuclear matrix and ribosomes.

Bacteria also have subcellular localizations that can be separated when the cell is fractionated. The most common localizations referred to include the cytoplasm, the cytoplasmic membrane (also referred to as the inner membrane in Gram-negative bacteria), the cell wall (which is usually thicker in Gram-positive bacteria) and the extracellular environment. Most Gram-negative bacteria also contain an outer membrane and periplasmic space. Unlike eukaryotes, most bacteria contain no membrane-bound organelles, however there are some exceptions.

There, we used wolfpsort a subcellular localization predication soft to predict subcellular localization. Wolfpsort an updated version of PSORT/PSORT II for the prediction of eukaryotic sequences.

Protein Complex

Protein complexes are key molecular entities that integrate multiple gene products to perform cellular functions. CORUM is a database that provides a manually curated repository of experimentally characterized protein complexes from mammalian organisms, mainly human (64%), mouse (16%) and rat (12%). The new CORUM 2.0 release encompasses 2837 protein complexes offering the largest and most comprehensive publicly available dataset of mammalian protein complexes. The CORUM dataset is built from 3198 different genes, representing approximately 16% of the protein coding genes in humans. Each protein complex is described by a protein complex name, subunit composition, function as well as the literature reference that characterizes the respective protein complex. Recent developments include mapping of functional annotation to Gene Ontology terms as well as cross-references to Entrez Gene identifiers.

3.3.2. Motif Analysis

Soft motif-x was used to analysis the model of sequences constituted with amino acids in specific positions of modifier-21-mers (10 amino acids upstream and downstream of the site) in all protein sequences. And all the database protein sequences were used as background database parameter, other parameters with default.

3.3.3. Functional Enrichment

Enrichment of Gene Ontology analysis

Proteins were classified by GO annotation into three categories: biological process, cellular compartment and molecular function. For each category, we used Functional Annotation Tool of DAVID Bioinformatics Resources 6.7 to identify enriched GO against the background of Homo sapiens. A two-tailed Fisher's exact test was employed to test the enrichment of the protein-containing IPI entries against all IPI proteins. Correction for multiple hypothesis testing was carried out using standard false discovery rate control methods. The GO with a corrected p-value < 0.05 is considered significant.

Enrichment of pathway analysis

Encyclopedia of Genes and Genomes (KEGG) database was used to identify enriched pathways by Functional Annotation Tool of DAVID against the background of Homo sapiens. A two-tailed Fisher's exact test was employed to test the enrichment of the protein-containing IPI entries against all IPI proteins. Correction for multiple hypothesis testing was carried out using standard false discovery rate control methods. The pathway with a corrected p-value < 0.05 was considered significant. These pathways were classified into hierarchical categories according to the KEGG website.

Enrichment of protein domain analysis

For each category proteins, InterPro (a resource that provides functional analysis of protein sequences by classifying them into families and predicting the presence of domains and important sites) database was researched using Functional Annotation Tool of DAVID against the background of Homo sapiens. A two-tailed Fisher's exact test was employed to test the enrichment of the protein-containing IPI entries against all IPI proteins. Correction for multiple

hypothesis testing was carried out using standard false discovery rate control methods and domains with a corrected p-value < 0.05 were considered significant.

Enrichment of complex analysis

Manually curated CORUM protein complex database for human was used for protein complex analysis. Overrepresented complexes were identified using hypergeometric test for each category proteins. A two-tailed Fisher's exact test was employed to test the enrichment of the protein-containing SwissProt entries against all SwissPort human proteins. Correction for multiple hypothesis testing was carried out using standard false discovery rate control methods and complexes with a corrected p-value < 0.05 were considered significant.

3.3.4. Enrichment-based Clustering

All the protein categories obtained after enrichment were collated along with their P values, and then filtered for those categories which were at least enriched in one of the clusters with P value<0.05. This filtered P value matrix was transformed by the function $x = -\log10$ (P value). Finally these x values were z-transformed for each category. These z scores were then clustered by one-way hierarchical clustering (Euclidean distance, average linkage clustering) in Genesis. Cluster membership was visualized by a heat map using the "heatmap.2" function from the "gplots" R-package.
